# A New Saurolophine Dinosaur from the Latest Cretaceous of Far Eastern Russia

**DOI:** 10.1371/journal.pone.0036849

**Published:** 2012-05-30

**Authors:** Pascal Godefroit, Yuri L. Bolotsky, Pascaline Lauters

**Affiliations:** 1 Department of Palaeontology, Institut Royal des Sciences Naturelles de Belgique, Bruxelles, Belgium; 2 Institute of Geology and Nature Management, Far Eastern Branch of the Russian Academy of Sciences, Blagoveschensk, Russia; 3 Section of Anthropology, Université Libre de Bruxelles, Bruxelles, Belgium; State Natural History Museum, Germany

## Abstract

**Background:**

Four main dinosaur sites have been investigated in latest Cretaceous deposits from the Amur/Heilongjiang Region: Jiayin and Wulaga in China (Yuliangze Formation), Blagoveschensk and Kundur in Russia (Udurchukan Formation). More than 90% of the bones discovered in these localities belong to hollow-crested lambeosaurine saurolophids, but flat-headed saurolophines are also represented: *Kerberosaurus manakini* at Blagoveschensk and *Wulagasaurus dongi* at Wulaga.

**Methodology/Principal Findings:**

Herein we describe a new saurolophine dinosaur, *Kundurosaurus nagornyi* gen. et sp. nov., from the Udurchukan Formation (Maastrichtian) of Kundur, represented by disarticulated cranial and postcranial material. This new taxon is diagnosed by four autapomorphies.

**Conclusions/Significance:**

A phylogenetic analysis of saurolophines indicates that *Kundurosaurus nagornyi* is nested within a rather robust clade including *Edmontosaurus* spp., *Saurolophus* spp., and *Prosaurolophus maximus*, possibly as a sister-taxon for *Kerberosaurus manakini* also from the Udurchukan Formation of Far Eastern Russia. The high diversity and mosaic distribution of Maastrichtian hadrosaurid faunas in the Amur-Heilongjiang region are the result of a complex palaeogeographical history and imply that many independent hadrosaurid lineages dispersed without any problem between western America and eastern Asia at the end of the Cretaceous.

## Introduction

**Figure 1:**
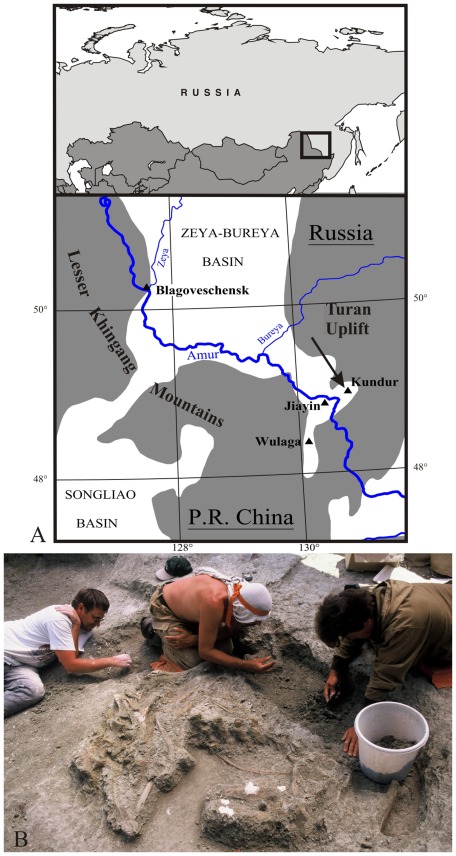
Maastrichtian (Late Cretaceous) dinosaur localities in the Amur/Heilongjiang Region. A: location of the main dinosaur sites (▴); the Kundur locality is indicated by an arrow and the grey zones indicate the uplifted areas. B: excavation of the Kundur locality in 2001.

Four rich dinosaur localities have been discovered in the Amur/Heilongjiang region of eastern Asia (Fig. 1A): Jiayin [1], [2] and Wulaga [3] in the Yuliangze Formation of northern Heilongjiang Province (China), Blagoveschensk [4], [5] and Kundur [6], [7], in the Udurchukan Formation of southern Amur Region (Russia). All these sites are located in the south-eastern part (‘Lower Zeya depression’) of the Zeya-Bureya sedimentary basin, near its borders with the adjacent uplifted areas: the Lesser Khingang Mountains and the Turan uplift. In the four sites, the dinosaur bones form large bonebeds extending over several hundreds of square metres [7], [8]. In each locality, the dinosaur fauna is largely dominated by lambeosaurine hadrosaurids [2]–[4], [6], but hadrosaurine (non-crested or solid-crested) hadrosaurids are also represented: *Kerberosaurus manakini* at Blagoveschensk [5] and *Wulagasaurus dongi* [3] at Wulaga.

The Kundur locality was discovered in 1990 by V.A. Nagorny (Far Eastern Institute of Mineral Resources, FEB RAS), who collected fossil bones in a road section along the Chita – Khabarovsk highway near the village of Kundur. He immediately sent his discoveries to Y. L. Bolotsky (Institute of Geology and Nature Management, FEB RAS). Large-scale excavations started at Kundur in 1999 (Fig. 1B). This dinosaur locality has yielded a nearly complete skeleton, several fragmentary skeletons and isolated bones of a new lambeosaurine hadrosaurid, *Olorotitan arharensis* [6], together with isolated bones and teeth belonging to theropods [9], nodosaurids [10], and lindholmemydid turtles [11]. The first multituberculate mammal fossil ever discovered in Russia was also described from Kundur locality [12].

The greatest part of the dinosaur material from Kundur, including the fossils described in the present paper, are included within massive, unsorted strata representing the deposits of ancient sediment gravity flows that originated from the uplifted areas at the borders of the Zeya-Bureya Basin. These gravity flows assured the concentration of dinosaur bones and carcasses as well as their quick burial. Such taphonomic conditions allowed the preservation of sub-complete hadrosaurid skeletons unearthed at the Kundur site [7].

The age of the Kundur locality is still subject to debates. Although the three sites belong to the same *Wodehouseia spinata* – *Aquilapollenites subtilis* palynozone, Markevich & Bugdaeva [13] date the Kundur and Jiayin dinosaur localities as Early Maastrichtian, whereas Blagoveschensk is dated as ‘middle’ Maastrichtian. The proposed ages are based on comparisons with other palynological assemblages in neighbouring basins. They assert that both the vegetation change and dinosaur extinction in the Russian Far East took place at the locally defined ‘middle’-Upper Maastrichtian boundary. Nevertheless, the pollen assemblage described in Kundur resembles the *Wodehouseia spinata* Assemblage Zone of the United States [7], [14], which is Late Maastrichtian in age [15], [16]. Consequently, it may be hypothesized that the Udurchukan and Yuliangze Formations are Late Maastrichtian in age, not Early or ‘middle’ Maastrichtian and that the observed dinosaur extinction and vegetation changes mark the Upper Maastrichtian – Paleocene boundary, not the 'middle' Maastrichtian – Upper Maastrichtian boundary, as proposed by the Russian colleagues. To close this debate, independent indicators (palaeontologic, geochronologic, or magnetostratigraphic) are yet to be found in the Maastrichtian deposits of the Amur-Heilongjiang Region.

Besides the abundant *Olorotitan arharensis* material, the Kundur locality has also yielded a partially articulated skull, a well-preserved pelvic girdle and numerous isolated bones belonging to a new saurolophine saurolophid. Because of the homogeneity of the recovered material, there is no reason to believe that more than one single saurolophine taxon lived in the Kundur area by latest Cretaceous time. The present paper describes this new saurolophine and discusses its phylogenetic, biostratigraphic and palaeogeographical significance.

## Materials and Methods

### Institutional Abbreviations

AENM, Amur Natural History Museum of the Institute of Geology and Nature Management, FEB RAS, Blagoveschensk, Russia. AMNH, American Museum of Natural History, New York, USA. MOR, Museum of the Rockies, Bozeman, USA. PIN, Paleontological Institute of the Russian Academy of Sciences, Moscow, Russia. ROM, Royal Ontario Museum, Toronto, Canada. TMM, Texas Memorial Museum, Austin, USA. TMP, Royal Tyrrell Museum of Palaeontology, Drumheller, Canada. USNM, United States National Museum, Washington D.C., USA. ZPAL, Institute of Paleobiology, Polish Academy of Sciences, Warsaw, Poland.

### Ethic Statements

According to the legislation of the Russian Federation, all necessary permits were obtained for the described field studies from the Land Resources Department of the Amurskaya Oblast’ (Russian Federation) and from the Far Eastern Branch of the Russian Academy of Sciences.

## Results

### Systematic Paleontology

Dinosauria Owen, 1842 [17].

Ornithischia Seeley, 1887 [18].

Saurolophidae Brown, 1914 [19] *sensu* Prieto-Márquez, 2010 [20].

Saurolophinae Brown, 1914 [19] *sensu* Prieto-Márquez, 2010 [20].

Kundurosaurus nagornyi gen. et sp. nov.

#### ZooBank life science identifier (LSID) for genus

**u**rn:lsid:zoobank.org:act: 4A699D11-A13E-4739-AF63-F1166A181057.

#### Zoobank LSID for species

urn:lsid:zoobank.org:act:F0B031EB-B21B-4AEC-B129-6F4BB5DC7F0C.

#### Holotype

AENM 2/921, a partial, disarticulated skull.

#### Referred specimens

AENM 2/45, 2/46, jugals; AENM 2/83, 2/84, 2/86, maxillae; AENM 2/57, 2/58, nasals; AENM 2/48, postorbital; AENM 2/19, quadrate; AENM 2/121, 2/928 partial braincases; AEHM 2/846, 2/902, dentaries; AENM 2/906, scapula; AENM 2/913, sternal; AENM 2/117, 2/903, 2/907, 2/908, humeri; AENM 2/905, ulna; AENM 2/904, radius; AENM 2/922, nearly complete pelvic girdle and associated sacral elements.

#### Specific diagnosis (as for genus by monotypy)

Saurolophinae characterized by the following autapomorphies: prominent and thick ridge on the lateral side of the nasal that borders caudally the circumnasal depression and invades the caudal plate of the nasal; caudal buttress of proximal head of scapula oriented quite laterally, parallel to the pseudoacromial process; preacetabular process of ilium straight and only moderately deflected ventrally (angle of ventral deflection: 160°): it does not reach the level of the plane formed by the bases of the iliac and pubic peduncles; axis of the postacetabular process strongly twisted along its length, so that its lateral side progressively faces dorsolaterally.

#### Locality and horizon

Kundur (N49°04′57.5″/E130°51′34.1″), Amur Region, Far Eastern Russia. Udurchukan Formation (*Wodehouseia spinata - Aquilapollenites subtilis* palynozone), ?Late Maastrichtian, Late Cretaceous.

#### Etymology

*Kundurosaurus*, from Kundur, the type-locality, and the transliterated Greek *sauros* (lizard); *nagornyi*, in honour of V.A. Nagorny (Far Eastern Institute of Mineral Resources, FEB RAS), who discovered the Kundur locality.

### Osteological Description

**Figure 2:**
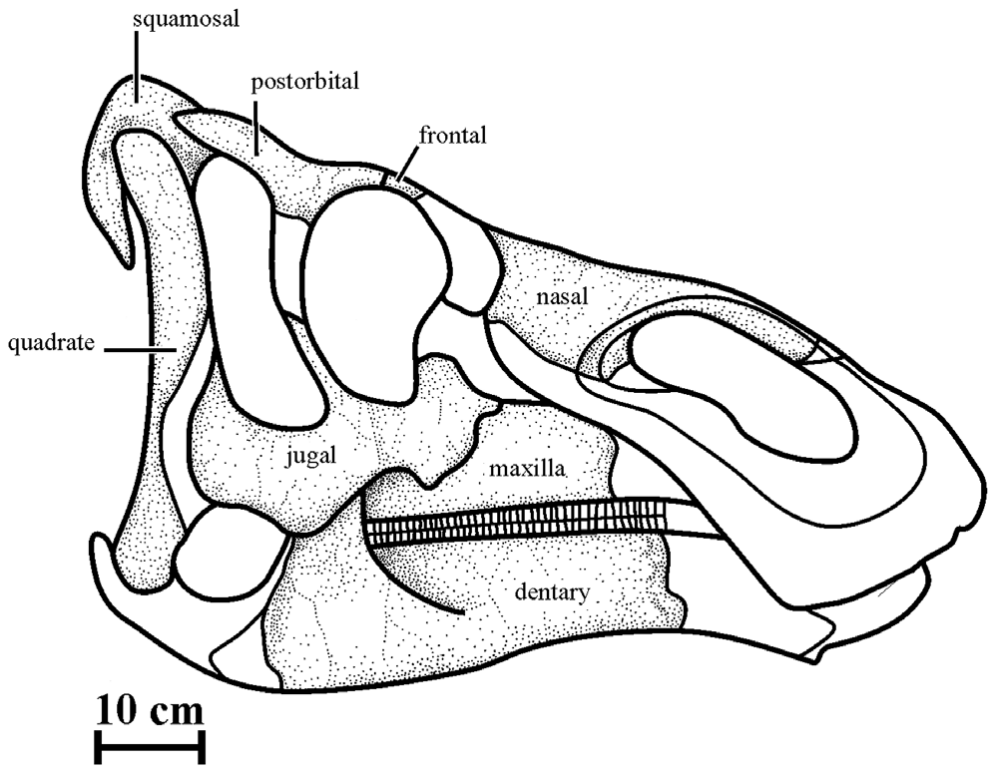
Reconstruction of the skull of *Kundurosaurus nagornyi* gen. et sp. nov. The dotted areas indicate the portions preserved in the Kundur fossil material.

Measurements on the holotype and referred specimens are available as online supplementary information (Table S1). The description of the skull of *Kundurosaurus nagornyi* is partly based on the holotype AENM 2/921. It is completed by the description of bones found at the same level, but that may belong to other individuals. A reconstitution of the skull of *Kundurosaurus nagornyi* is proposed in Fig. 2. Disarticulated forelimb elements with typical saurolophine morphology have been found in the same layer as the *Kundurosaurus nagornyi* holotype skull. They can be easily distinguished from the equivalent bones of *Olorotitan arharensis* discovered in the same locality. Because there is no indication that more than one hadrosaurine taxon lived in the Kundur area by Late Maastrichtian time, those fossils with hadrosaurine morphology are tentatively attributed to *Kundurosaurus nagornyi*.

**Figure 3:**
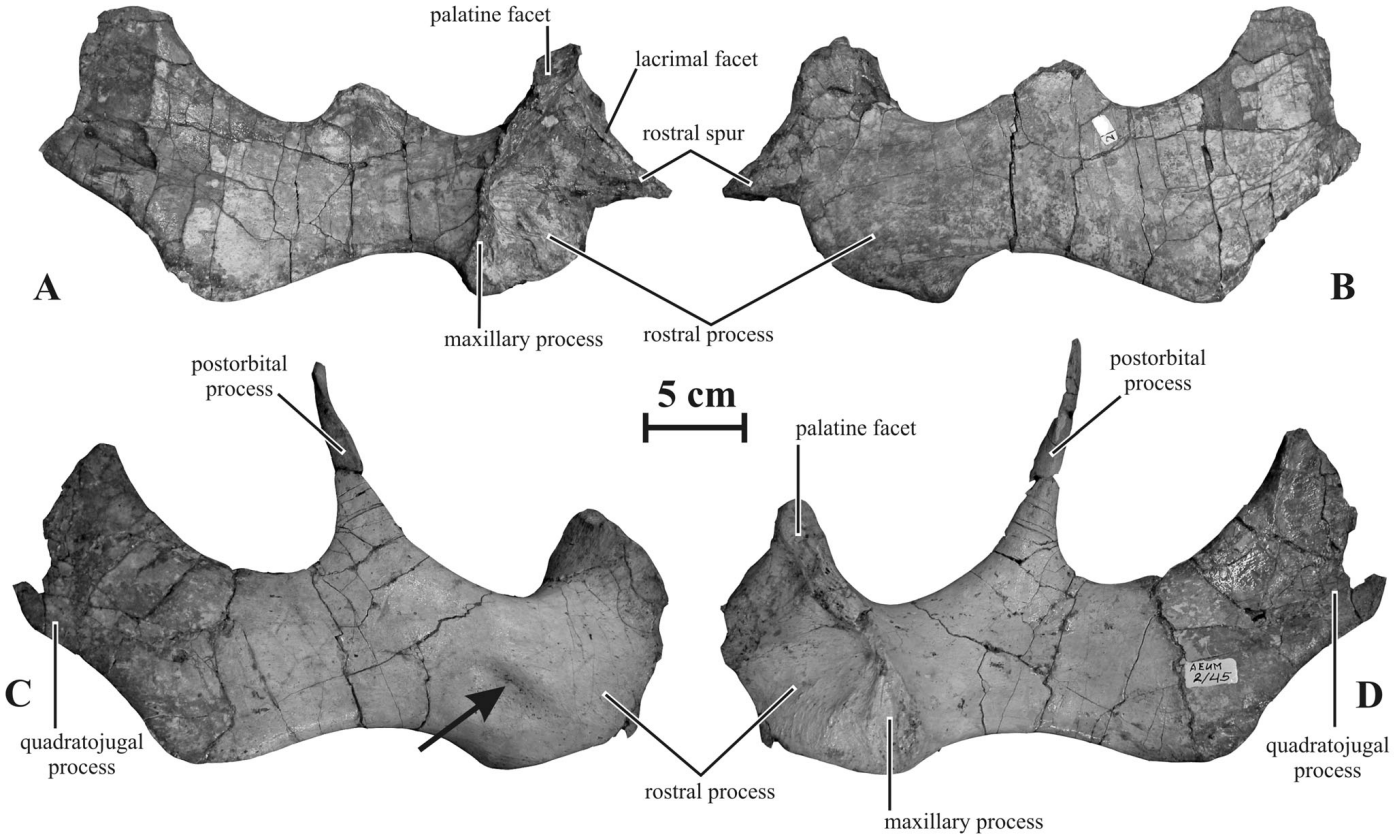
Jugals of *Kundurosaurus nagornyi* gen. et sp. nov. Left jugal (AENM 2/921-2) in medial (A) and lateral (B) views. Right jugal (AENM 2/45) in lateral (C) and medial (D) views.

Jugal (AENM 2/45, 2/46, 2/921-2). The jugal (Fig. 3) is robust and rostrocaudally elongated. The morphology of its rostral process closely resembles that of *Gryposaurus notabilis* (TMP 80.22.1). In lateral view, it is asymmetrical and strongly upturned. It forms a short, robust and sharply-pointed triangular spur. Contrary to *Maiasaura peeblesorum* and *Brachylophosaurus canadensis*, this triangular spur is very asymmetrical and not centered, but set above mid-height of the rostral plate. The dorsal border of the triangular spur forms a laterally-everted lip, the lacrimal facet (Fig. 3A). Its ventral border is nearly horizontal, so that the rostral process looks notched in lateral view. The medial side of the rostral process forms a large and deeply excavated maxillary facet. An elevated vertical crest limits it caudally. The ventral part of this crest forms an elliptical and slightly concave plateau, the maxillary process. The dorsal part of the crest is widened to form the elliptical palatine facet. The postorbital process is long, very slender and elliptical in cross-section. It ascends at nearly a 90° angle. Its dorsal portion forms a flattened rostral facet for articulation with the postorbital. The quadratojugal process raises caudodorsally at nearly the same angle as the postorbital process. It is mediolaterally thin and appears more robust dorsoventrally than in *Gryposaurus* spp. Its ventral margin is slightly concave. At the angle between the quadratojugal process and the main body of the jugal, a flange is developed, so that the dorsoventral depth of the jugal from the ventral border of the infratemporal fenestra to the ventral edge of the flange is about 1.5 times as high as the minimum dorsoventral depth of the rostral segment of the jugal, between the rostral and postorbital processes. The quadratojugal facet forms a well-marked depressed area on the medial side of the quadratojugal process. The lateral side of AENM 2/45 forms an elliptical depression, probably of pathological origin, between the rostral and postorbital processes (Fig. 3C). It must also be noted that the ventral curvature is highly variable in the jugals referred to as *Kundurosaurus nagornyi*. It could therefore be argued that several saurolophine taxa are represented in the Kundur bonebed. However, the ventral curvature seems intraspecifically variable in saurolophine, directly depending on several factors such as the development of the rostral process, the ventral flange, and the rostral constriction. For that reason, we consider that the degree of curvature of the ventral margin of the jugal is not a good diagnostic character and that it must be cautiously considered in phylogenetic analyses.

#### Maxilla (AENM 2/83, 2/84, 2/86)

**Figure 4:**
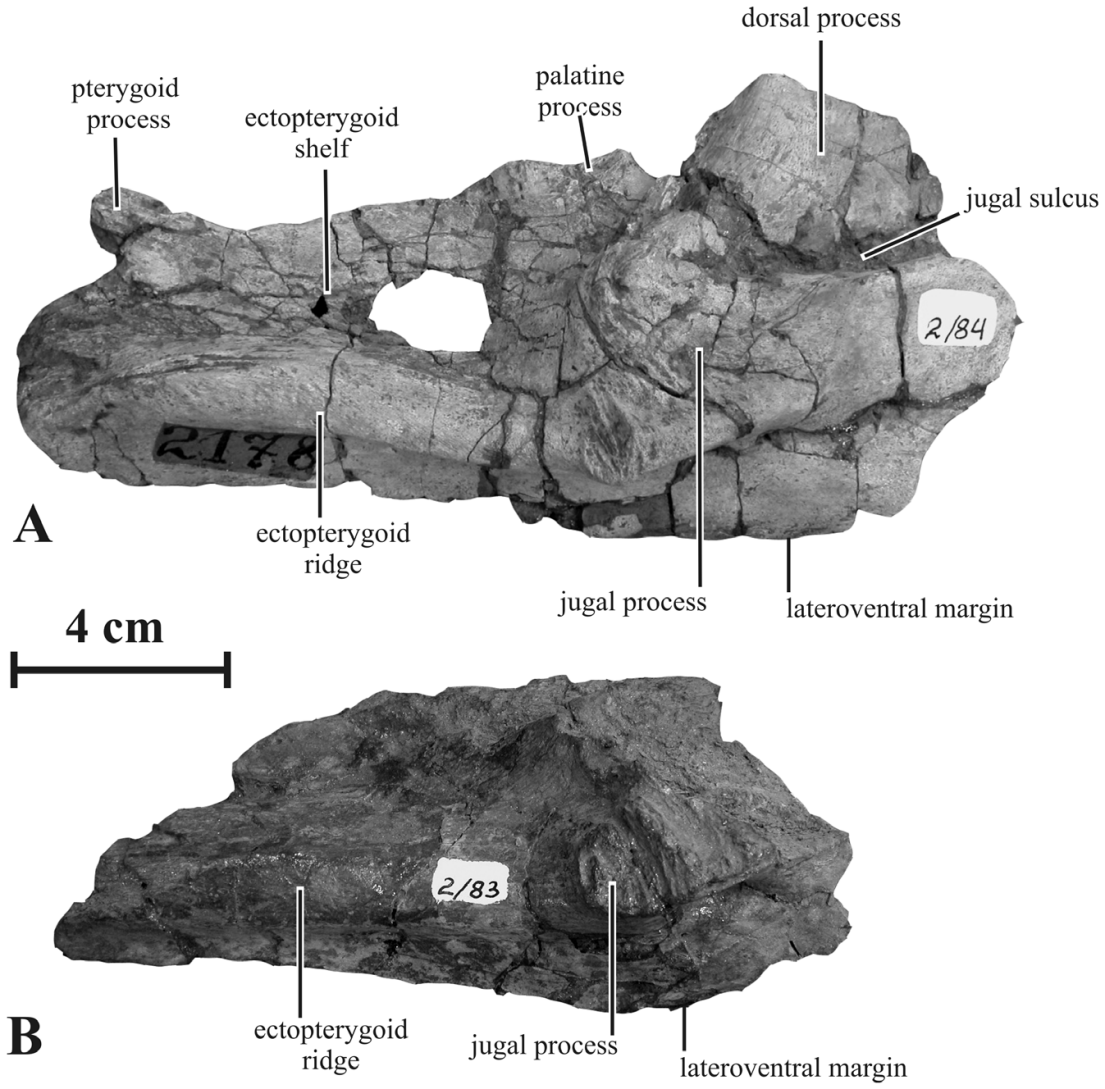
Right maxillae of *Kundurosaurus nagornyi* gen. et sp. nov., in lateral view. A: AENM 2/84. B: AENM 2/83.

Maxillae referred to as *Kundurosaurus nagornyi* are incompletely preserved, lacking their rostral and medial portions (Fig. 4). However, they display a characteristic saurolophine morphology: although it is broken, the dorsal process appears proportionally low and the caudal portion of the bone is particularly long and robust. The dorsal process appears less rostrocaudally long and robust than in *Kerberosaurus manakini*, but it can also be interpreted as an ontogenetic character. Caudoventrally to the dorsal process, the lateral side of the maxilla forms a wide, prominent, and concave jugal process that faces slightly dorsally. The jugal process is prolonged rostrodorsally by a deep horizontal sulcus, which received the ventral border of the rostral spur of the jugal (Fig. 4A). Such a sulcus is also figured in *Edmontosaurus* [21]. Below the jugal process, the ventral margin of the maxilla is very convex in lateral view. Caudally to the dorsal process, the palatine process forms an elongated concave facet along the dorsolateral border of the maxilla. This situation contrasts with the hook-like palatine process described in *Kerberosaurus manakini* [5]. Between the dorsal and palatine processes, an oblique groove communicates with the excavated caudomedial surface of the dorsal process. Ventrally to the jugal process, the lateral side of the maxilla is pierced by four large foramina. The ectopterygoid ridge is prominent and nearly horizontal; only its caudal part is deflected ventrally. The ectopterygoid shelf is long, wide and dorsoventrally concave. The caudal part of the dorsal border of the maxilla has a large hook-like pterygoid process.

#### Nasal (AENM 2/57, 2/58)

**Figure 5:**
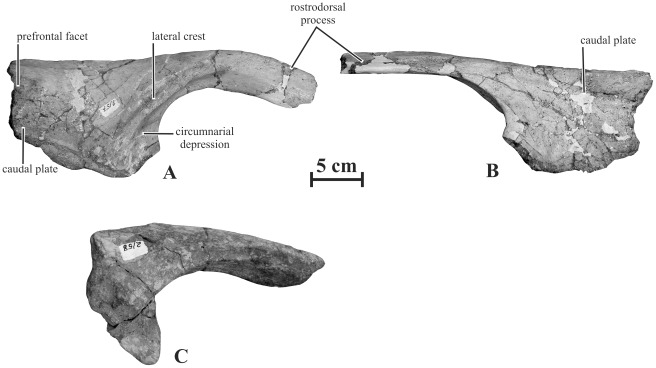
Right nasals of *Kundurosaurus nagornyi* gen. et sp. nov. AENM 2/57 in lateral (A) and medial (B) views. AENM 2/58 in lateral view (C).

The nasal of *Kundurosaurus nagornyi* is formed by a wide caudal plate and by a robust rostrodorsal process that forms the dorsal and caudal margins of the external nares (Fig. 5). It is much more robust and more curved downwards than in *Kerberosaurus manakini* [5]. However, it is not as strongly arched as in *Gryposaurus notabilis* (ROM 873) or in *Gryposaurus monumentensis* [22]. Its medial side is flat, where it contacted the paired process. The caudal part of its lateral side bears a strong flattened crest that marks the dorsal and caudal limits of the circumnarial depression. Contrary to *Kerberosaurus*, this crest does not closely follow the margin of the external naris, but it invades the caudal plate. The circumnarial depression is not invaginated at this level, as frequently observed in *Edmontosaurus* and *Saurolophus* adult specimens [20]. The caudal plate of the nasal is proportionally shorter than in *Kerberosaurus manakini* [5]: the distance between the rostral point of the articulation with the prefrontal and the caudal point of the external naris is shorter than the height of the plate. The caudal margin of the dorsoventrally convex lateral side of the caudal plate bears a large depressed triangular facet for articulation with the prefrontal. A similar prefrontal facet can also be observed in *Gryposaurus latidens* [23]. The ventral border is depressed along its whole length for articulation with the premaxilla and the lacrimal. The medial side of the caudal plate is very concave where it enclosed the nasal cavity. The rostroventral portion of the nasal plate is broken off, but it apparently participated in the caudoventral margin of the external naris, as e.g. observed in *Gryposaurus* spp. [22] and *Brachylophosaurus canadensis* [24].

#### Postorbital (AENM 2/48, 2/921-6)

**Figure 6:**
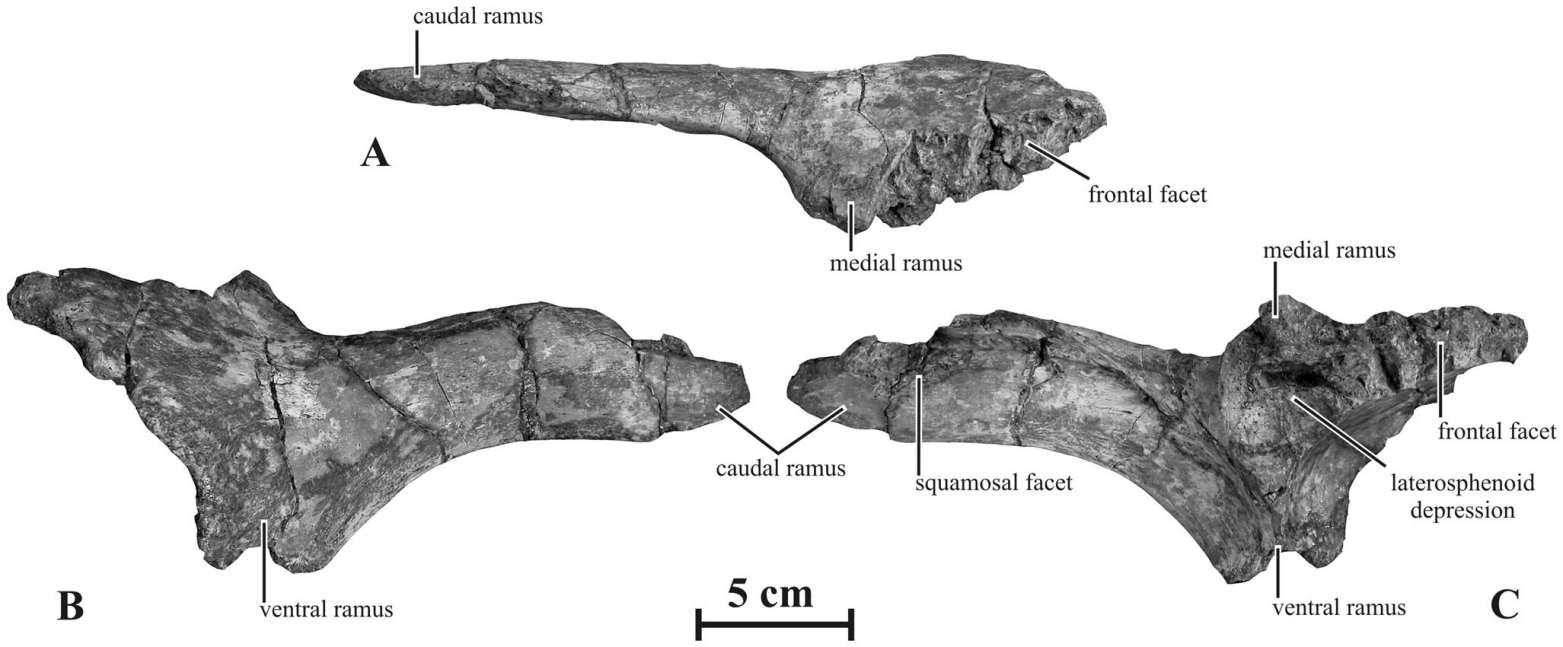
Left postorbital (AENM 2/921-6) of *Kundurosaurus nagornyi* gen. et sp. nov., in dorsal (A), lateral (B), and medial (C) views.

The postorbital is a triradiate bone formed by a medial, a caudal and a ventral ramus oriented at about 90° from each other (Fig. 6). It is low and rostrocaudally elongated. In lateral view, the dorsal surface of the postorbital above the jugal process is markedly depressed, as also observed in *Saurolophus osborni* and *Saurolophus angustirostris* [20]. The medial ramus, which forms the rostral corner of the supratemporal fenestra, is particularly stout. The articular surface for the frontal forms a very large notch, with a thick and persillate border for intimate contacts (Fig. 6A). The caudal ramus is elongated, mesiolaterally compressed and slightly convex upwards. It is distinctly longer than in *Gryposaurus monumentensis* [22], but more slender than in *Edmontosaurus* spp. [21]. On its medial side, a wide and elongated groove that progressively deepens rostrally marks the contact with the rostral ramus of the squamosal. The ventral ramus of the postorbital is broken off in the available specimens. The internal orbital surface does not form any enlarged pouch as in *Edmontosaurus* spp. At the junction between the three rami, a large pocket-like depression received the postorbital process of the laterosphenoid in a synovial joint (Fig. 6C). The dorsolateral orbital rim of the postorbital is very rugose. This feature suggests that the hadrosaurid postorbital results from the fusion of the ‘true’ postorbital with a small supraorbital II [25].

#### Frontal (AENM 2/921-7)

**Figure 7:**
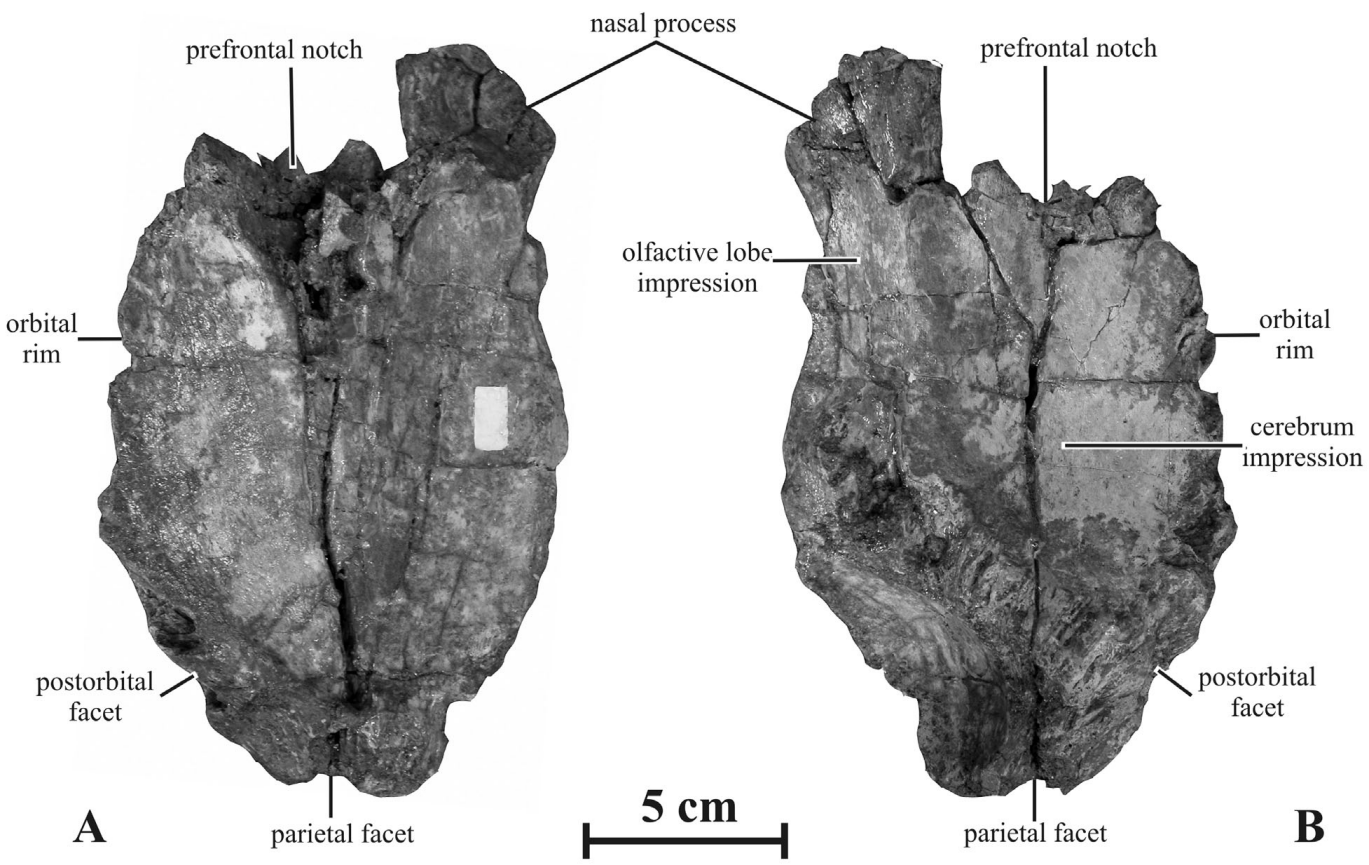
Left frontal (AENM 2/921-7) of *Kundurosaurus nagornyi* gen. et sp. nov., in dorsal (A) and ventral (B) views.

The frontal of *Kundurosaurus nagornyi* is massive and particularly wide (Fig. 7). This condition contrasts with the narrow frontals of *Kerberosaurus manakini* [5]. Its dorsal surface is essentially flat; however, the bone is slightly more elevated medially, so that it looks slightly concave mediolaterally. The frontal is thick caudally and forms a persillate and interdigitate contact with the parietal. The caudolateral side of the frontal is also thickened and roughened for interdigitate contact with the postorbital. The rostrolateral side of the frontal is deeply notched by the articular surface for the prefrontal. Between the articular surfaces for the prefrontal and the postorbital, the lateral margin of the frontal participated in the dorsal margin of the orbit. The rostromedial corner of the frontal forms a narrow depressed process that supported the dorsal part of the rostral plate of the nasal. Caudally to the nasal process, the medial margin of the frontal is slightly notched, suggesting that small medial processes of the paired nasals inserted between the midline of the frontals, as observed in *Gryposaurus* spp. [22], [23]. Caudally to this notch, the medial margin of the frontal is particularly thin: this is the place where a frontal-nasal fontanella was described in several juvenile hadrosaurines and basal hadrosauroids [25]–[28]. In ventral view, the caudomedial portion of the frontal is deeply excavated by the rostral part of the cerebrum. Around this area, strong rugosities mark the contact area with the laterosphenoid and orbitosphenoid portions of the braincase (Fig. 7B). Rostromedially, the ventral side of the frontal bears an elongate encephalic impression, probably for the olfactive lobe of the brain.

#### Squamosal (AENM 2/921-5)

**Figure 8:**
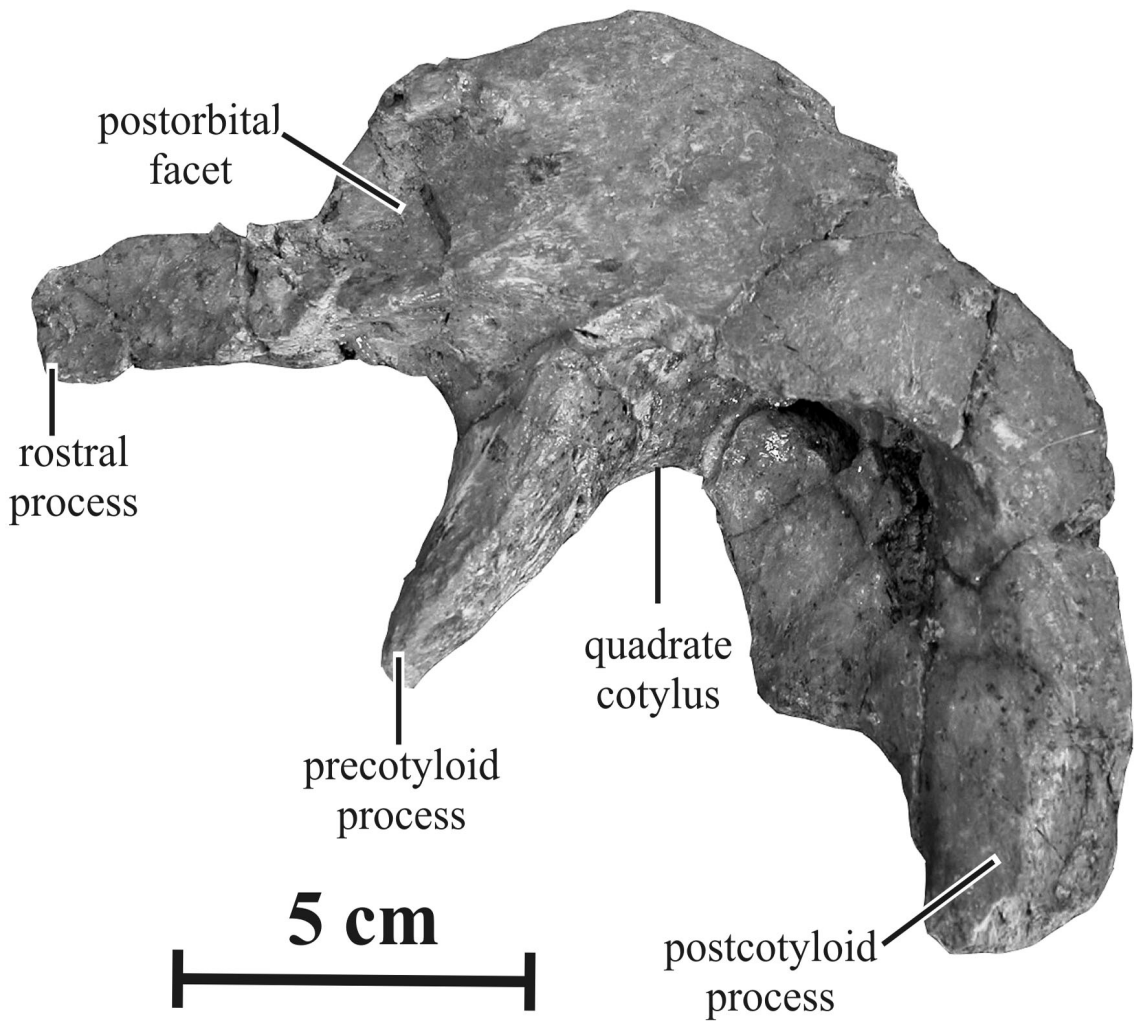
Left squamosal (AENM 2/921-5) of *Kundurosaurus nagornyi* gen. et sp. nov., in lateral view.

The squamosal of *Kundurosaurus nagornyi* has a typical saurolophine morphology, with a low lateral wall above the quadrate cotylus (Fig. 8). The rostral process of the squamosal is mediolaterally compressed and its lateral side is deeply excavated for reception of the caudal ramus of the postorbital. The precotyloid process is robust and triangular in cross-section. Although it is incomplete, it is strikingly longer than the rostrocaudal width of the quadrate cotylus or the dorsal head of quadrate; it is, in any case, proportionally longer than in *Maiasaura* and *Brachylophosaurus* [24]. The precotyloid fossa is poorly marked on the lateral side of the squamosal. The postcotyloid process is also robust and mediolaterally compressed. Both the pre- and postcotyloid processes limit a very deep quadrate cotylus.

#### Quadrate (AENM 2/19, 2/921-3, 2/921-4)

**Figure 9:**
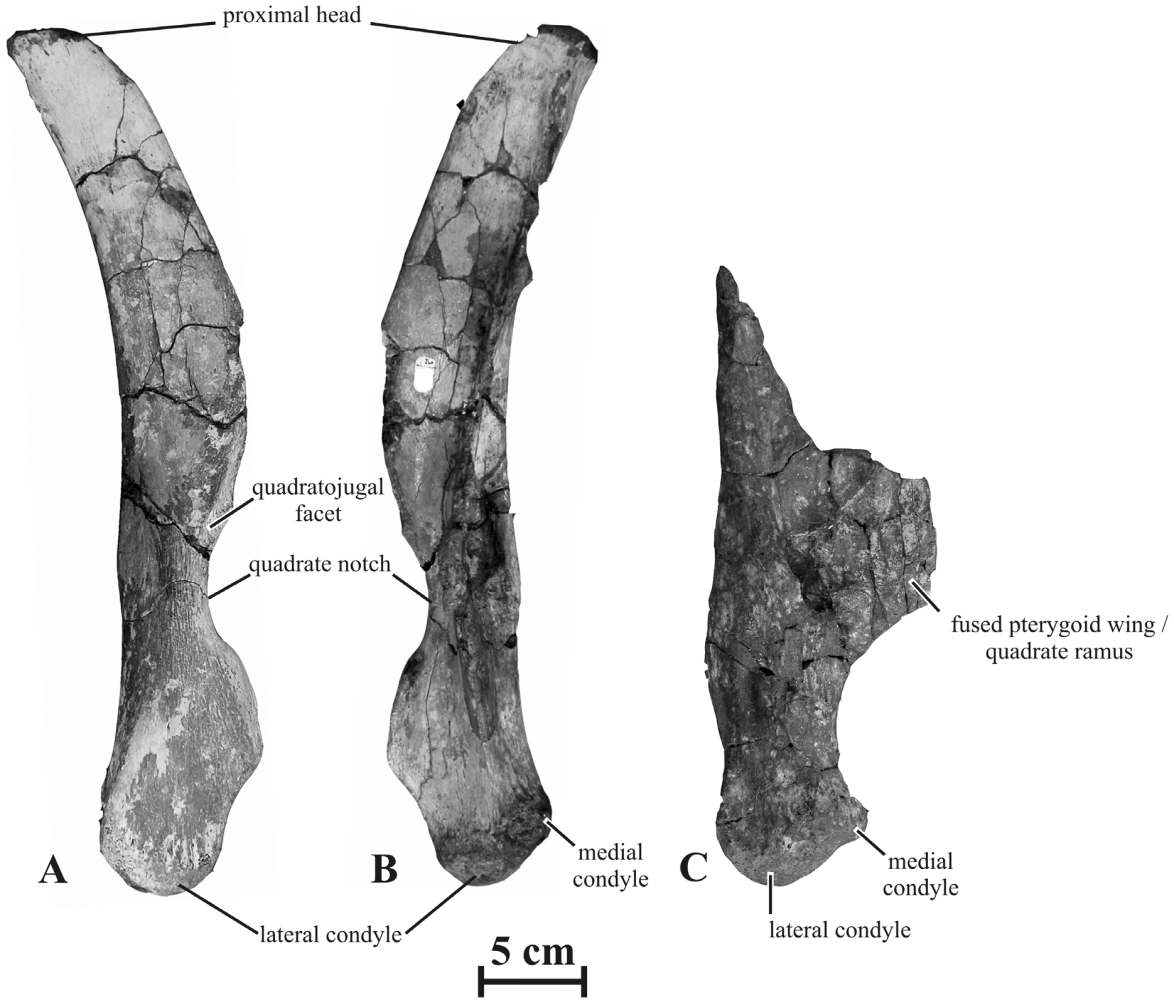
Quadrates of *Kundurosaurus nagornyi* gen. et sp. nov. Right quadrate (AENM 2/921-3) in lateral (A) and medial (B) views. C: left quadrate (AENM 2/921-3) in caudal view.

The quadrate of *Kundurosaurus nagornyi* is high, moderately bowed caudally, and relatively narrow in lateral view (Fig. 9). It is more robust than in *Kerberosaurus manakini* [5]. The ratio ‘height of the quadrate/length of the jugal’  = 1.2 in the holotype, suggesting that the skull was proportionally high dorsoventrally, like in *Gryposaurus* spp. [22]. The proximal quadrate head of AENM 2/921-3 is rounded, sub-triangular in cross-section and mediolaterally flattened. The quadrate notch appears proportionally shorter, but deeper than in *Kerberosaurus*. As it is usual in saurolophines, the midpoint of the quadrate notch is located ventral to the mid-height of the quadrate: the ratio between the distance from the mid-height of the notch to the quadrate height and the height of the bone is 0.7, similar to the condition observed in *Edmontosaurus* ssp. [20]. The lateral border around the quadrate notch is depressed around its whole height, indicating that it was completely closed in life by the quadratojugal. As it is usual in saurolophids, the distal head of the quadrate is composed of a large rounded lateral condyle that articulated in the surangular part of the mandibular glenoid, and of a smaller medial condyle, set more dorsally and that fitted into the articular component of the mandibular glenoid. The greatest part of the pterygoid wing is destroyed on both quadrates of the holotype specimen. On the left specimen (AENM 2/921-4), the quadrate ramus of the pterygoid is partly preserved and pathologically fused to the rostral part of the pterygoid wing and to the medial part of the quadrate body, so that the respective limits of the bones cannot be discerned (Fig. 9C). Because the quadrate is not deformed, it is unlikely that those bones were diagenetically compressed against each other.

#### Parietal (AENM 2/121, 2/921-8)

**Figure 10:**
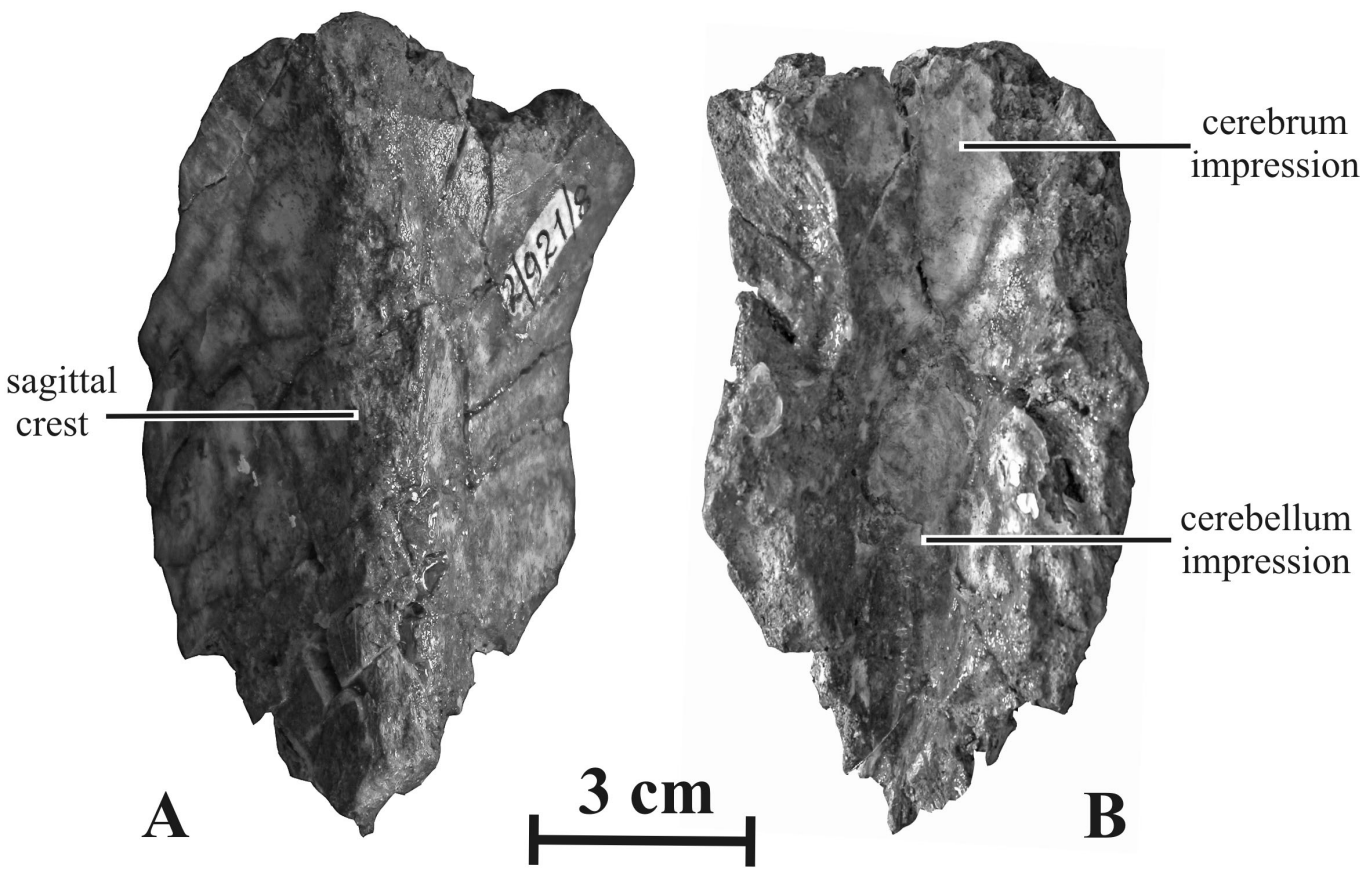
Parietal (AEHN 2/921-8) of *Kundurosaurus nagornyi* gen. et sp. nov., in dorsal (A) and ventral (B) views.

**Figure 11:**
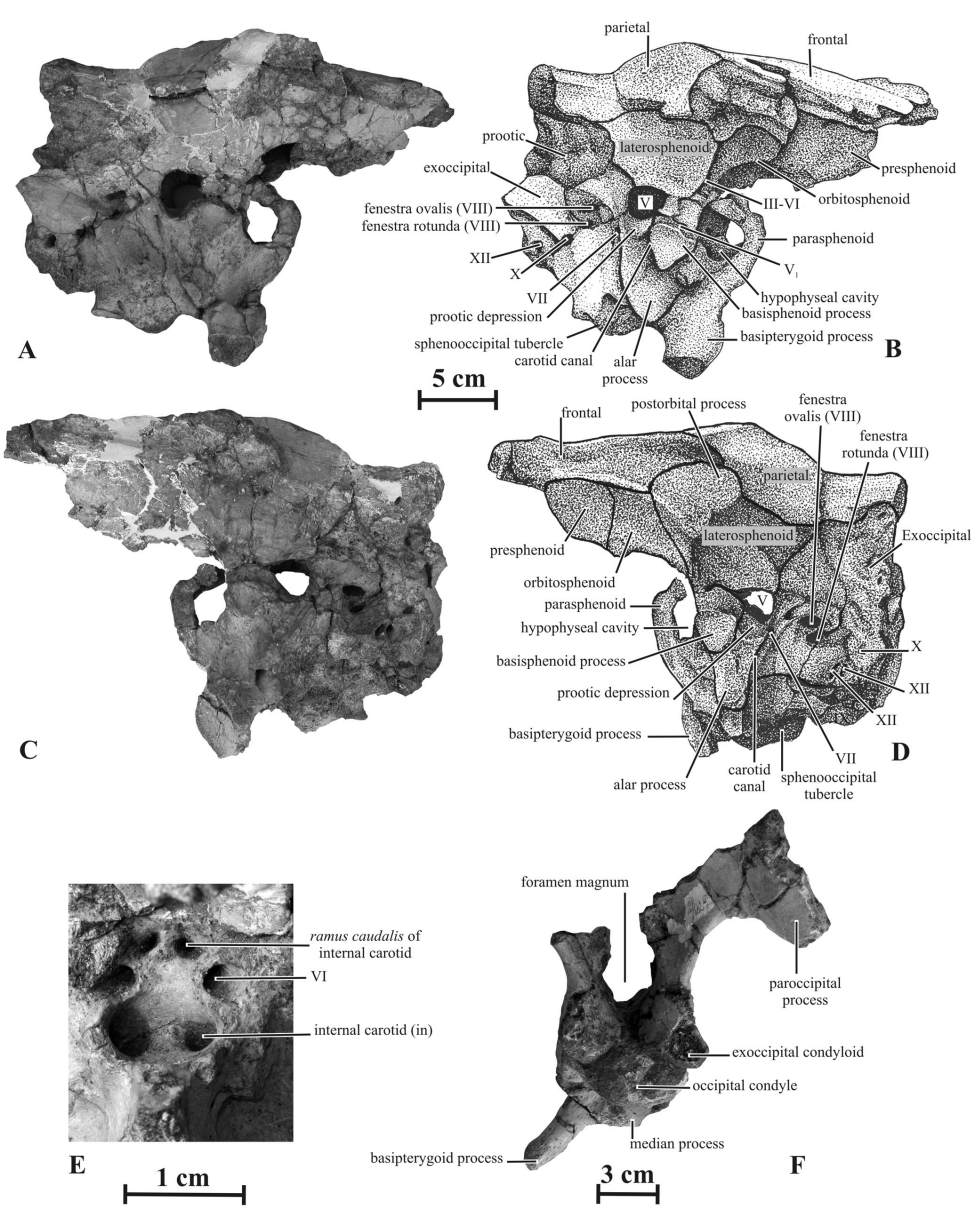
Braincase (AENM 2/121) of *Kundurosaurus nagornyi* gen. et sp. nov., in right (A, B) and left (C, D) lateral views, close-up of the hypophyseal cavity (E). F: caudal view of the braincase (AENM 2/928).

The parietal of *Kundurosaurus nagornyi* is long and transversely narrow, with a ‘length/minimal width’ ratio >3 (Fig. 10–11). Along nearly its whole length, the parietal has a strong sagittal crest. Far rostrally, this crest flattens and widens to form a lozenge-shaped surface. Although it is incompletely preserved, the rostral margin of the parietal is apparently not depressed around the contact area with the frontals as in *Kerberosaurus manakini* [5]. In ventral view, the impression area for the cerebellum is narrow, but deep. The rostral impression for the distal part of the cerebrum is wider, but shallower.

#### Prootic (AENM 2/121, 2/921-1)

The prootic of *Kundurosaurus nagornyi* is particularly massive (Fig. 11A-D). Its caudodorsal ramus, which covered the rostral part of the exoccipital-opisthotic, is wide and stout. The rostral margin of the auditory foramen notches the caudoventral portion of the prootic, whereas the caudal margin of the trigeminal nerve (V) notches its rostral part. Below this latter foramen, the ventral part of the prootic is deeply excavated by a pocket-like depression. This pocket is separated from the trigeminal foramen by a horizontal ridge. This is the situation observed in *Kerberosaurus manakini* [5], but also in *Brachylophosaurus canadensis* (pers. obs.). In *Edmontosaurus* spp. and *Saurolophus* spp., on the other hand, this pocket is not developed, but a vertical groove, which probably transmitted the ramus mandibularis (V3), runs from this foramen along the lateral surface of the prootic. Between the notches for the auditory foramen and the trigeminal nerve, the lateral wall of the prootic is pierced by two smaller foramina. The caudodorsal foramen transmitted the hyomandibularis branch of the facial nerve (VII), whereas the cranioventral foramen transmitted the palatinus branch of the same nerve. A long and narrow groove runs from the latter foramen along the lateral side of the prootic. The prootic forms a ventrally directed flange that covers the lateral side of the basisphenoid. This flange has a strong vertical ridge, in continuity with the alar process of the basisphenoid.

#### Laterosphenoid (AENM 2/121, 2/921-1)

The laterosphenoid of *Kundurosaurus nagornyi* (Fig. 11A-D) is a stout bone formed by three processes. The prootic process, which contacts the parietal dorsally and covers the prootic ventrally, forms a wide, triangular and caudally-directed wing. The basisphenoid process forms a ventrally-directed foot that covers the basisphenoid and the rostrodorsal part of the ventral flange of the prootic. The angle between the prootic and the basisphenoid processes forms the rostral margin of the foramen for the trigeminal nerve. From this notch, a wide and deep groove extends rostrally along the lateral side of the laterosphenoid, indicating the rostral passage of the deep *ramus ophthalmicus* of the trigeminal nerve (V_1_). The postorbital process of the laterosphenoid is elongated and stout. From the tip of the postorbital process to the basisphenoid process, the lateral side of the laterosphenoid has a regularly rounded crest marking the separation between the orbit and the supratemporal fenestra.

#### Orbitosphenoid (AENM 2/121, 2/921-1)

This bone participates in the rostral part of the lateral wall of the braincase and in the greatest part of the incomplete interorbital septum (Fig. 11 A-D). Its dorsal border contacts the frontal, its caudal border the laterosphenoid, its ventral border the parasphenoid, and its rostral border the presphenoid. A common foramen for the oculomotor (III) and abducens (VI) nerves is located between the parasphenoid and the orbitosphenoid, at the caudoventral corner of the latter.

#### Presphenoid (AENM 2/121, 2/921-1)

Only a portion of the presphenoid is preserved in these specimens (Fig. 11A-D), but it does not provide any valuable information.

#### Basioccipital (AENM 2/121, 2/921-1, 2/928)

In caudal view, the basioccipital is kidney-shaped (Fig. 11F). It appears rostrocaudally elongated, when compared with other advanced hadrosaurids (Fig. 11A-D). Two prominent tubercles, projecting lateroventrally from the basioccipital, form the caudal half of the sphenooccipital tubercles.

#### Basisphenoid (AENM 2/121, 2/921-1, 2/928)

The caudal part of the basisphenoid is developed into a pair of large processes, separated by a wide and deep fossa; these processes form the rostral part of the sphenooccipital tubercles. More rostrally, the stout basipterygoid processes diverge caudolaterally from the base of the basisphenoid at an angle of about 45° from the horizontal. A small median process projects caudoventrally from the caudal junction between both basipterygoid processes (Fig. 11F). The deep carotid canal extends obliquely along the dorsal part of the basipterygoid process. The alar process that concealed rostrally the carotid canal is broken off (Fig.11 A-D). The rostrodorsal surface of the basisphenoid is deeply excavated by the hypophyseal cavity. Two large foramina, which correspond to the entrance of the internal carotid arteries, open in the ventrocaudal part of the hypophyseal cavity (Fig. 11E). Two pairs of foramina are visible on the caudodorsal wall of this cavity: the ventrolateral pair corresponds to the passage for the abducens (VI) nerves, whereas the dorsomedial pair corresponds to the passage for ramus caudalis of the internal carotid artery [29].

#### Exoccipital (AENM 2/121, 2/928)

The exoccipitals are much eroded and damaged and the main interesting characters cannot be adequately distinguished. The exoccipital condyloid is large and is pierced by three foramina, successively. The oval vagus foramen (CN X) is the largest and is bordered ventrally by two smaller foramina interpreted as opening conducting branches of the hypoglossal nerve (CN XII) [30]. Rostrally to these foramina, a strong ridge extends obliquely along the lateral side of the condyloid. This crest is not developed in *Kerberosaurus manakini* [5]. In caudal view, the exoccipitals apparently formed an extended shelf that roofed the foramen magnum (Fig.11F), contrasting with the shorter shelf in *Maiasaura peeblesorum*, *Brachylophosaurus canadensis*, and *Wulagasaurus dongi* [3], [23].

#### Parasphenoid (AENM 2/921-1)

The parasphenoid is poorly preserved. It participates in the ventral margin of the large common opening for the occulomotor (III) and abducens (VI) nerves (Fig. 11A-D).

#### Dentary (AENM 2/846, 2/902)

**Figure 12:**
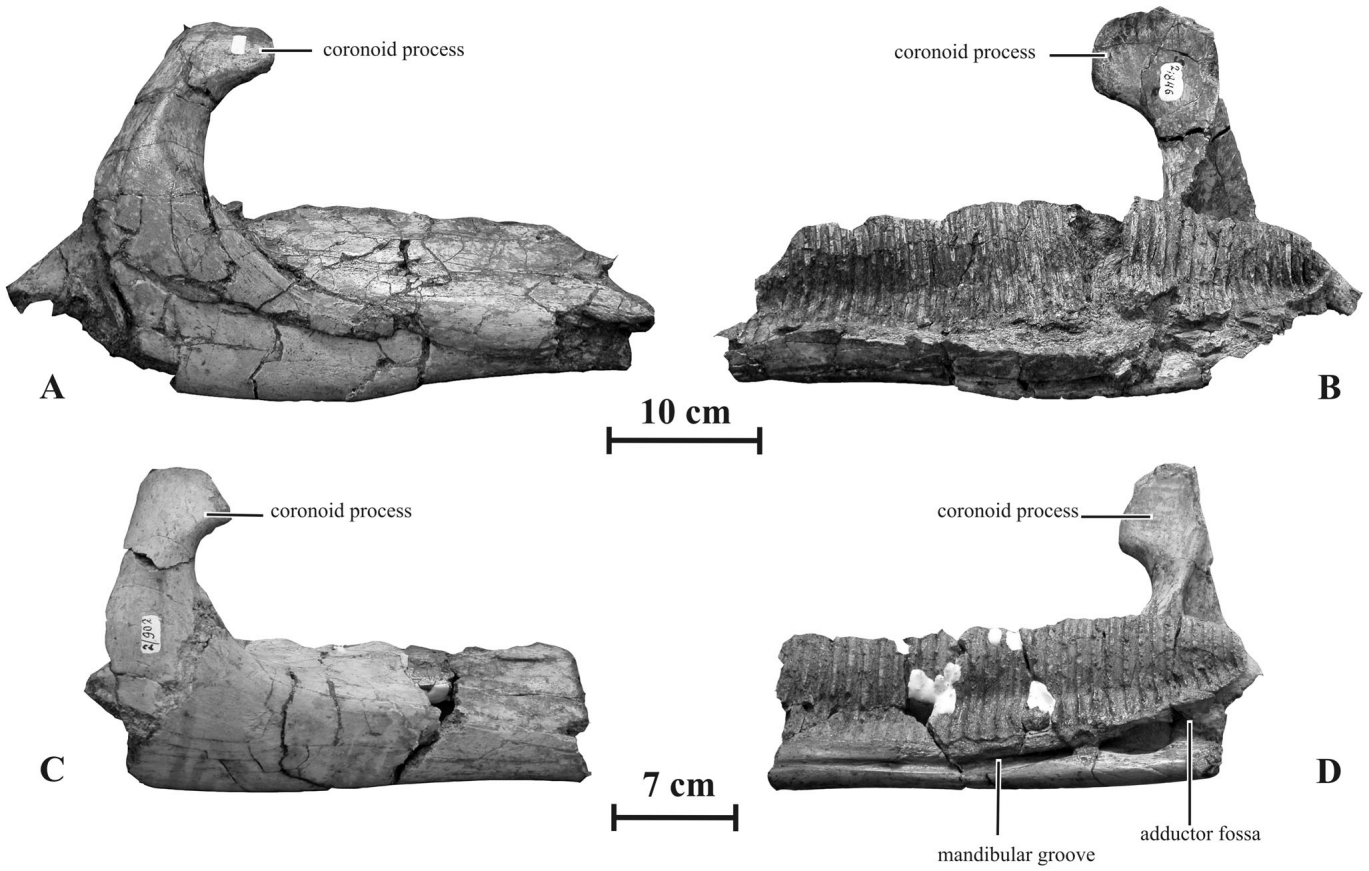
Dentaries of *Kundurosaurus nagornyi* gen. et sp. nov. A-B: AENM 2/846 in lateral (A) and medial (B) views. C-D: AENM 2/902 in lateral (C) and medial (D) views.

Two incomplete dentaries discovered in Kundur locality display significant differences with *Olorotitan* specimens from the same site, more closely resembling typical saurolophine dentaries (Fig. 12). They are therefore tentatively referred to as *Kundurosaurus nagornyi*. Unfortunately the diastema and the symphysis are not preserved in both specimens, and the dental battery is completely dissociated. The lateral side of the dentary is proportionally high and moderately convex dorsoventrally, and pierced by 5 or 6 sparsely distributed foramina. In AENM 2/846, the largest specimen, the dental battery fitted into more than 41 narrow parallel-sided alveolar grooves, visible in medial view (Fig. 12B). Viewed from above, the dentary ramus is perfectly straight. In lateral view, the ventral border of the dentary is also perfectly straight along the whole length of the dental battery area. The coronoid process is proportionally high and slender. The height of the coronoid process, taken between the apex of the process and the dorsal border of the dentary ramus, is greater than the maximal height of the dentary ramus. This character can of course be correlated with the important height of the quadrate and with the high proportions of the skull as a whole. The apex of the coronoid process is slightly inclined rostrally as usually observed in saurolophids. Its lateral side is convex both rostro-caudally and dorso-ventrally, whereas its medial side is slightly concave. In AENM 2/846, the dental battery extends caudally well beyond the level of the caudal border of the apex of the coronoid process (Fig. 12B). Under the coronoid process, the dentary is deeply excavated by the rostral portion of the adductor fossa; it extends rostrally as a deep mandibular groove (Fig. 12D).

#### Neuroanatomy (AENM 2/121)

**Figure 13:**
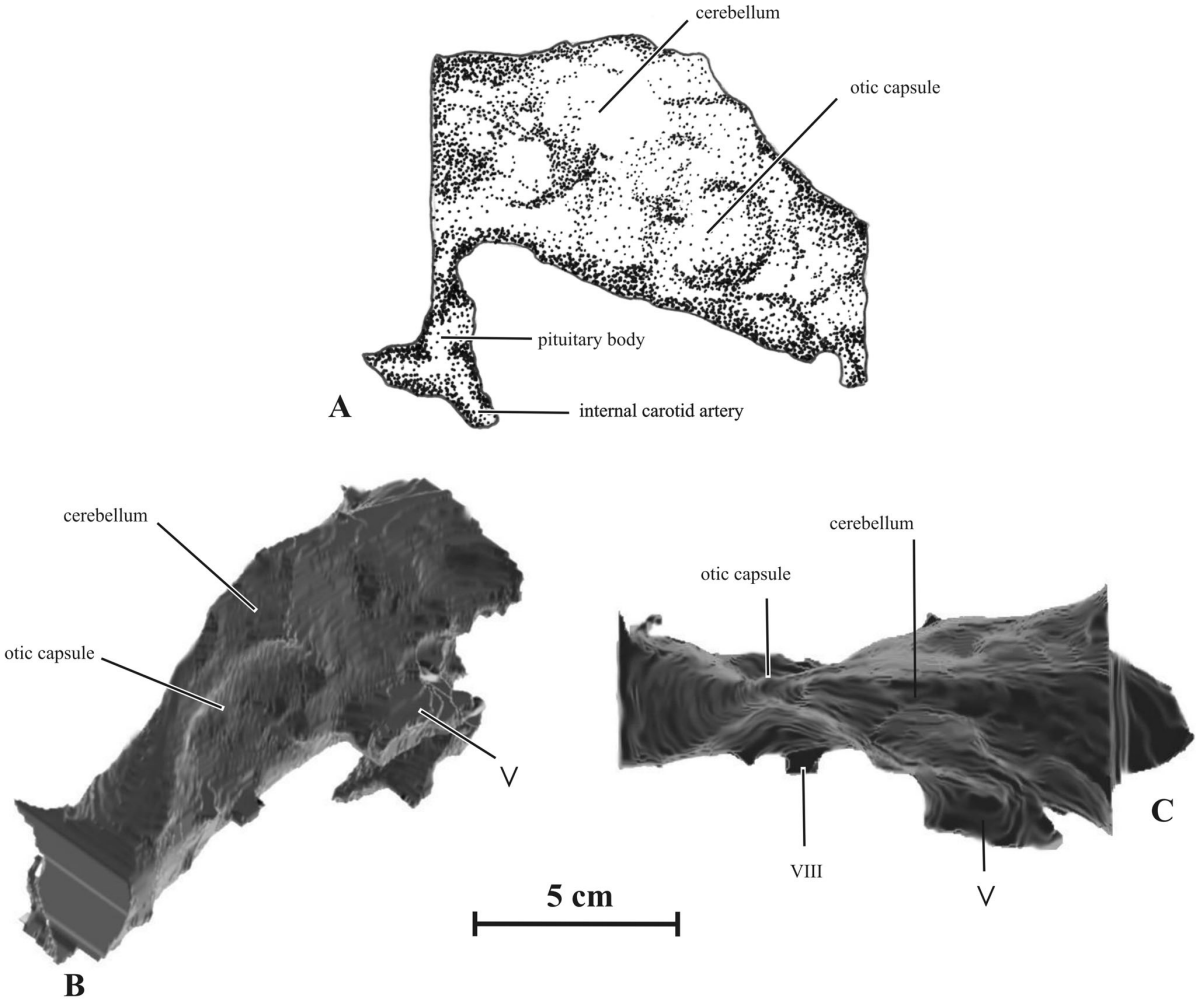
Endocranial reconstruction of *Kundurosaurus nagornyi* gen. et sp. nov. (AENM 2/121). A: drawing of the left lateral view. B: rear 3/4 view, reconstructed from CT scan. C: dorsal view, reconstructed from CT scan.

The braincase of *Kundurosaurus nagornyi* was scanned in the coronal plane, in three millimeter slice thickness with 1.5 millimeter overlap using Siemens Emition scanner in the Amur Region Hospital in Blagoveschensk. Selection and reconstruction were made in transverse plane using ArteCore from the VisiCore Suite. Different views of the reconstruction are presented in Fig. 13. The purpose of this work is not to describe the cranial nerves but the overall brain morphology of *Kundurosaurus nagornyi*. The resolution of the scanner did not allow reconstructing finite features like nerve foramina or semi-circular canals. The braincase was incomplete and therefore endocranial reconstruction was restricted to the posterior part of the brain, just behind the cerebral hemispheres.

The endocranial reconstitution is 115.29 mm long and 72.03 mm high at its largest dimensions. It is 63.58 mm at its largest point but due to the lack of cerebral hemispheres we can assess that the complete brain was larger. The volume of the reconstruction is 151 cm^3^.

The major divisions are distinct, although the precise limits are not discernible. The midbrain is constricted and slightly triangular in transverse section. The cerebellum was tight in transverse section and marks the highest point of the brain. The upper limit of the brain decreases rapidly after this point. The pituitary body is incomplete but large. Large internal carotid arteries enter it posterolaterally. The constriction behind the cerebellum is particularly visible in a dorsal view (Fig. 13C). This constriction is formed by the otic mass marking the position of the semi-circular canals. The cast of the medulla region is oval in transverse section, being slightly higher than wide. The brain shows no sign of pontine flexure.

Comparison with other endocranial casts from the literature reveals that the brain of *Kundurosaurus nagornyi* resembles that of other saurolophines [29]–[31]. It shares a lot of similarities with North American *Gryposaurus* endocasts [29]. It is distinguished from non-hadrosaurian ornithopod by the enlarged cerebrum and the absence of the pontine flexure [30]. Unfortunately the incompleteness of the braincase did not allow us to observe some characteristics like the expansion of the cerebrum or the size of the olfactory tracts.

#### Scapula (AENM 2/906)

**Figure 14:**
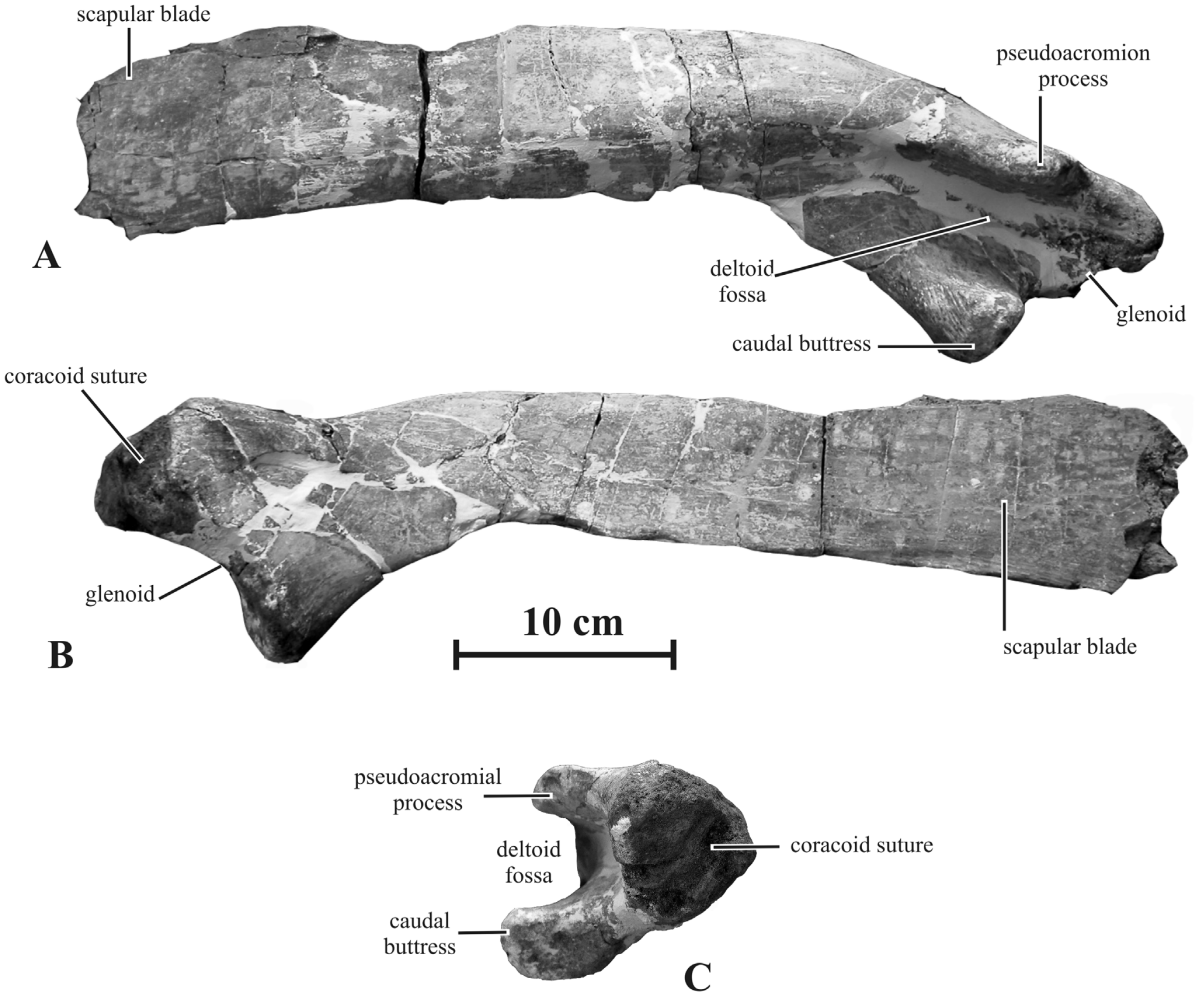
Right scapula (AENM 2/906) of *Kundurosaurus nagornyi* gen. et sp. nov., in lateral (A), medial (B), and ventral (C) views.

The scapula of *Kundurosaurus nagornyi* closely resembles that of *Gryposaurus notabilis* [32]. The proximal head is dorsoventrally low, but mediolaterally thick (Fig. 14C). The coracoid suture is broad, sub-triangular, slightly concave and very rough. The pseudoacromial process is strongly developed and oriented quite laterally, as usually observed in saurolophines [20]. It extends caudally as a rounded deltoid ridge that progressively fuses with the dorsolateral aspect of the scapular blade (Fig. 14A). Ventrally to the coracoid suture, the glenoid forms a large crescent-like depression, supported caudally by a prominent buttress from the ventral border of the scapula. Like the pseudoacromial process, this protuberance is oriented quite laterally. Consequently, the deltoid fossa, limited by the parallel pseudoacromial process and the caudal buttress, appears narrow but very deep and U-shaped (Fig. 14C). This lateral orientation of the caudal buttress is unusual in saurolophids: the caudal buttress is usually oriented ventrally to ventrolaterally. Although it is not completely preserved, the scapular blade appears mediolaterally thick and dorsoventrally low (Fig. 14A-B).

#### Sternal (AENM 2/911, 2/913)

**Figure 15:**
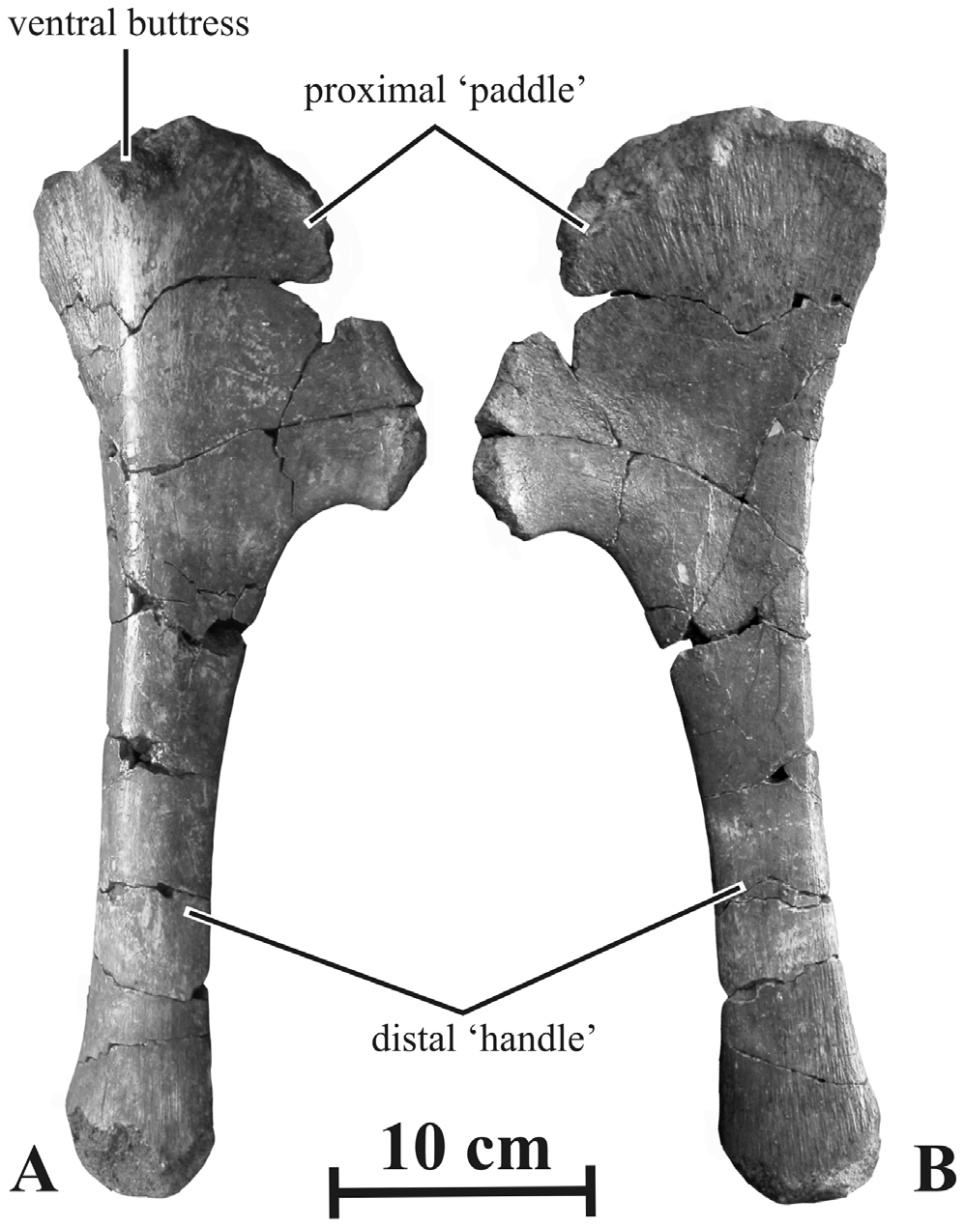
Right sternal (AENM 2/913) of *Kundurosaurus nagornyi* gen. et sp. nov., in ventral (A) and dorsal (B) views.

As it is usual in saurolophids, the sternal is formed by a paddle-like expanded proximal region located at the end of an elongated handle-like segment (Fig. 15). The proximal ‘paddle’ is much shorter than the distal ‘handle’, as in other saurolophines [33]. The ‘paddle’ is fan-like. Its dorsal side is slightly concave, whereas its ventral side is markedly convex. Its cranial border is very rough, indicating the presence of a cartilaginous cap in life. From its dorsal border, the ventral side of the paddle bears a prominent buttress, also figured in *Edmontosaurus annectens* [31]. The dorsal side of the paddle has numerous longitudinal striations, starting from the cranial border of the bone. The ‘handle’ is long and robust. Its ventral side is convex, whereas its dorsal side is flat. Its distal end is slightly expanded and has longitudinal striations on both sides.

#### Humerus (AENM 2/117, 2/903, 2/907, 2/908)

**Figure 16:**
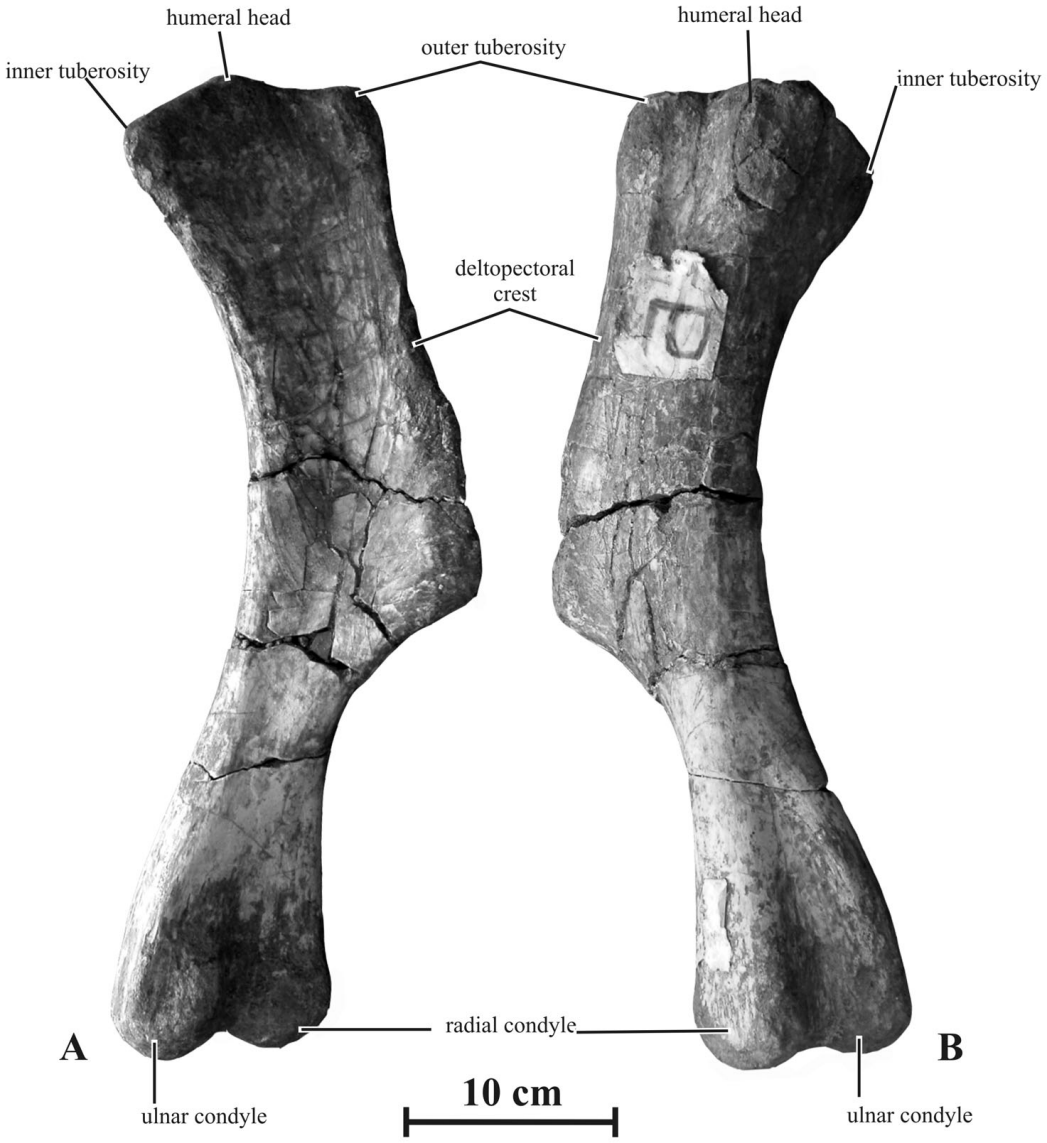
Left humerus (AENM 2/908) of *Kundurosaurus nagornyi* gen. et sp. nov., in cranial (A) and caudal (B) views.

Humeri tentatively referred to as *Kundurosaurus nagornyi* are rather robust when compared with those of other saurolophines such as *Edmontosaurus* spp. (Fig. 16). The articular head is globular and supported by a short buttress on the caudal side of the bone; it is separated from the outer tuberosity by a sulcus, but appears to be continuous with the inner tuberosity. The cranial side of the humerus forms a regularly concave bicipital sulcus. From the inner tuberosity, the medial side of the humerus is regularly concave. From the outer tuberosity, the deltopectoral crest extends craniolaterally down below the mid-point of the bone. It is not particularly wide and its border is straight to slightly concave. The distal portion of the humerus is slightly twisted outwards. The ulnar condyle is more prominent and extends more distally than the radial condyle. The intercondylar groove is equally developed along both sides of the bone.

#### Ulna (AENM 2/905)

Two ulna and radius morphotypes, a robust one and a gracile one, can be distinguished within the Kundur material. A rather gracile ulna was found associated with *Olorotitan* holotype. Although the size of this ulna corresponds with the general size of the holotype, it cannot be definitely asserted that it belongs to this specimen, because it was not found in connection with the humerus, but close to the head. On the other hand, associated robust right radius and ulna were found close to the *Kundurosaurus nagornyi* holotype skull. Here also, in spite of corresponding size and preservation, it cannot be definitely asserted that they belong to the same specimen. However, we have decided to tentatively assign those robust ulna and radius to *Kundurosaurus nagornyi*, pending the discovery of more complete specimens that would confirm or invalidate this association.

**Figure 17:**
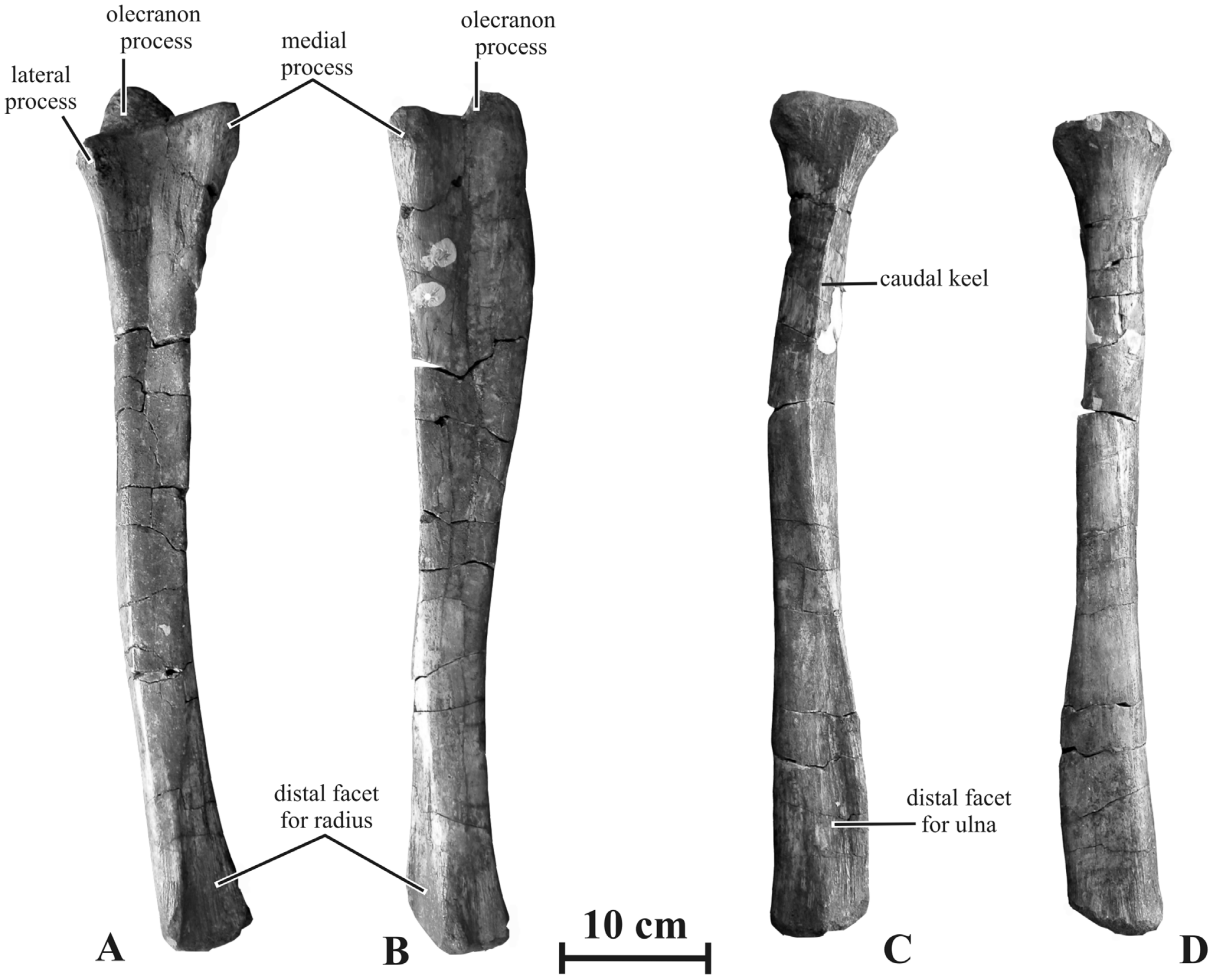
Forearm of *Kundurosaurus nagornyi* gen. et sp. nov. A-B: right ulna (AENM 2/905) in cranial (A) and medial (B) views. C-D: right radius (AENM 2/904) in caudal (C) and cranial (D) views.

The ulna of *Kundurosaurus nagornyi* is robust, like that of *Gryposaurus incurvimanus* [32] and that of *Gryposaurus notabilis* [34]. In cranial view, this bone is distinctly curved medially. It is slightly sigmoid in medial or lateral view: the proximal end is convex caudally, whereas the distal part is convex cranially (Fig. 17 A-B). The olecranon process is prominent, more developed, in any case than in the gracile morphotype. The medial proximal process is particularly high and robust, whereas the lateral one is distinctly lower and thinner. Between both processes, the cranial border of the ulna forms a deep and wide U-shaped triangular depression against which the proximal part of the radius articulated; longitudinal striations indicate strong ligamentous attachment with the radius. Under this area, the body of the ulna is craniocaudally high. It remains triangular in cross section along its whole length. The ulna progressively tapers distally. Its distal end is rounded and laterally compressed. The large triangular articular surface for the distal end of the radius faces craniomedially; a well-developed crest along the distal end of the ulna borders it laterally and it also bears strong longitudinal striations.

#### Radius (AENM 2/904)

The radius referred to as *Kundurosaurus nagornyi* is robust, as also observed in *Gryposaurus incurvimanus* [32] and that of *Gryposaurus notabilis* [34]. It is nearly perfectly straight (Fig. 17C-D). The proximal end of the radius is well expanded, resembling the top of a Doric column in cranial view; its cranial side is slightly convex, whereas its caudal side is flattened where it articulated with the proximal part of the ulna. At some distance from the proximal end, the caudal side of the radius forms a strong keel-like prominence that fits into the U-shaped depression on the cranial side of the ulna. Longitudinal striations indicate strong ligamentous attachment of the proximal head of the radius with the ulna. The distal end of the radius is mediolaterally much expanded, as also observed in *Gryposaurus notabilis* [34]. Its flattened caudolateral side forms a wide, strongly striated, triangular surface, which fitted against the distal part of the ulna. A strong lateral ridge limits this surface.

#### Ilium (AENM 2/922-6R, 2/922-7L)

The following description is based on a nearly complete pelvic girdle, in connection with sacral elements, found a few metres from the holotype skull. However, although it is tentatively referred to as *Kundurosaurus nagornyi*, there is no direct evidence that it belongs to the same individual as the holotype skull.

**Figure 18:**
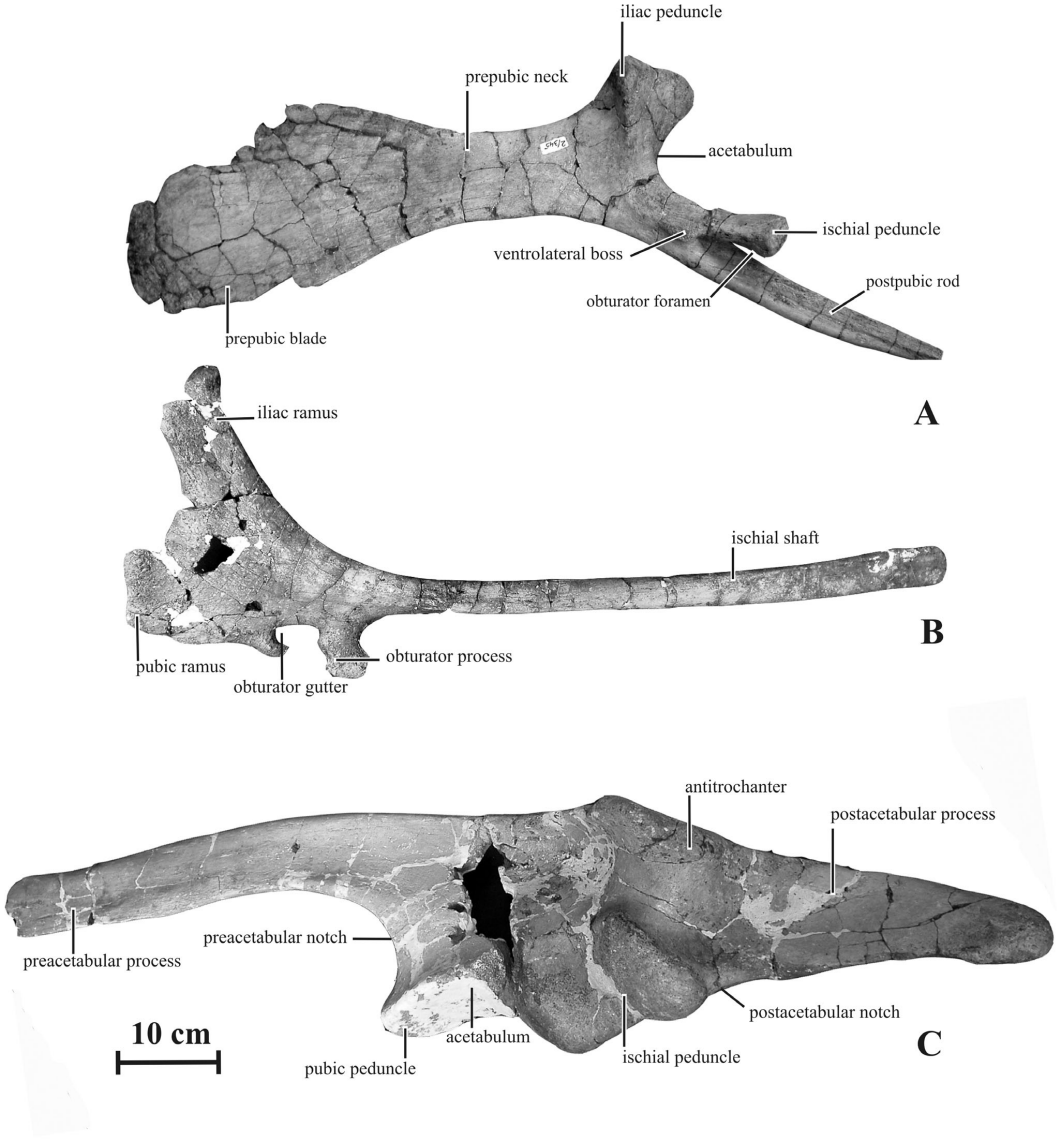
Pelvic girdle of *Kundurosaurus nagornyi* gen. et sp. nov. A: left pubis (AENM 2/922-5L) in lateral view. B: left ischium (AENM 2/922-3L) in lateral view. C: left ilium (AENM 2/922-7L) in lateral view.

**Figure 19:**
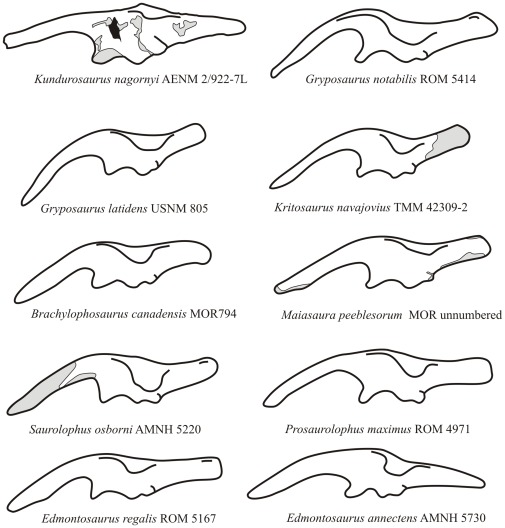
Ilium of *Kundurosaurus nagornyi* gen. et sp. nov., compared to other hadrosaurine ilia. Modified from [47].

The preacetabular process of the ilium of *Kundurosaurus nagornyi* forms a long and tapering projection from the craniodorsal edge of the iliac blade. It is straight and only moderately deflected ventrally. With an angle of ventral deflection of 160°, it does not reach the level of the plane formed by the bases of the iliac and pubic peduncles (Fig. 18C). In other saurolophines, on the other hand, the rostral point of preacetabular process is usually located at the level of or below this plane and the angle of ventral deflection is less than 150° (Fig. 19). The lateral side of the preacetabular process is perfectly flat. Its dorsal edge is very thickened and rounded, whereas its ventral edge is sharper. The caudal half of its medial side has, at about the dorsal third of its height, a strong carina. The main blade of the ilium is not very high. Its dorsal edge is sigmoid and thickened. At the level of the ischial peduncle, its dorsolateral border is folded laterally to form a prominent and roughened antitrochanter, nearly symmetrical in lateral view. The ventral extension of the antitrochanter is different on the left and right ilia, although they clearly belong to the same individual: although it extends lateroventrally between half and three quarters of the dorsoventral depth of the right ilium, it remains limited on the dorsal quarter of the dorsoventral depth of the left ilium. The supraacetabular process is also longer on the right ilium, although it is extremely difficult to quantify this character because the cranial and caudal ends of the process gradually merge with the dorsal margin of the ilium. It means that characters related to the development of the supraacetabular process must be cautiously considered in phylogenetic analyses. A strong ridge thickens medially the dorsal part of the main blade of the ilium, in continuity with that on the medial side of the preacetabular process. It fuses caudally with the dorsal border of the ilium, at the level of the ischial peduncle. The preacetabular notch is well developed and rather open, because of the slight ventral deflection of the preacetabular process. The pubic peduncle is relatively short, not very massive. The iliac portion of the acetabulum is rather deep and slightly asymmetrical. The ischial peduncle is elongated craniocaudally and laterally prominent. Its articular surface faces caudoventrally and is formed by two sub-rectangular protrusions separated by a well-marked depression. The postacetabular notch is only slightly marked. The postacetabular process is particularly long (around 90% of the length of the preacetabular process) and sub-rectangular in shape. Its dorsal border is thick mediolaterally, whereas its ventral border is sharp. Whereas the lateral side of the postacetabular process is perfectly flat, its medial side bears a strong rounded oblique ridge. The postacetabular process consequently looks triangular in cross-section. The axis of the postacetabular process is strongly twisted along its length, so that its lateral side progressively faces dorsolaterally. It is usually more vertical in other saurolophines. The dorsal margin of the postacetabular process is caudodorsally oriented, as it is usual in saurolophids, rising dorsally relative to the acetabular margin.

#### Pubis (AENM 2/922-4R, 2/922-5L)

The prepubic blade is ellipsoidal and craniocaudally longer than dorsoventrally high, resembling the condition encountered in *Maiasaura peeblesorum* and *Brachylophosaurus canadensis* [35]. It is less strongly deflected ventrally than in *Gryposaurus notabilis* (ROM 764). The prepubic neck is more contracted in *Kundurosaurus nagornyi* than in *Brachylophosaurus* [35]. The prepubic neck is longer than the prepubic blade, as in *Edmontosaurus* spp., but it remains more robust than in the latter [31]. The iliac peduncle is prominent and robust; a strong, vertical and roughened ridge along its lateral side limits rostrally the acetabular surface of the bone. A well-marked, triangular and striated surface on the medial side of the iliac peduncle reveals a close contact with one of the cranialmost sacral ribs. The ischial peduncle is long and its articular surface with the ischium is expanded and rounded. The proximal part of the ischial peduncle bears a well-marked ventrolateral boss, also described in *Brachylophosaurus* [35]. The development of this protuberance appears highly variable in the *Amurosaurus riabinini* specimens discovered in Blagoveschensk locality, probably reflecting ontogenetic variation. For that reason, the presence or absence of this character is not retained in the phylogenetic analysis presented herein (contra [20]). The postpubic rod is short, robust, mediolaterally compressed and gently curved. Together with the ischial peduncle, it limits a deep obturator foramen.

#### Ischium (AENM 2/922-2R, 2/922-3L)

The ischial shaft is slender, slightly curved and rod-like; the distal end tapers in a rounded point (Fig. 18B). The expanded cranial region of the ischium is not parallel to the parasagittal plane, but tilts a few degrees laterally. The iliac ramus is subrectangular and projects craniodorsally; its dorsal articular process is slightly expanded both mediolaterally and dorsoventrally and sub-ellipsoidal in cross section. The pubic ramus is more slender and less differentiated than the iliac ramus. It projects anteriorly and is very compressed mediolaterally. The articular facet for the pubis is sub-rectangular in cross section. The pubic ramus is slightly concave laterally and convex medially. Numerous striations are found extending craniocaudally across the lateral side of the pubic ramus, especially on its ventral portion. The obturator process is well developed, projecting ventrally lower than the pubic ramus. Its ventral border is expanded and closely contacted the pubic bar. It is prolonged caudally as a carina along the medioventral side of the ischial shaft. The obturator process and the pubic ramus limit an ovoid and ventrally-open obturator gutter. This gutter is closed ventrally, thus forming a foramen in *Saurolophus osborni* [36], in several specimens of *Saurolophus angustirostris* (ZPAL MgDI/159 and MgDI/169) and in *Brachylophosaurus canadensis* (MOR 794). However, it cannot be excluded that this character is ontogenetic amongst hadrosaurines.

#### Sacral vertebrae (AENM 2/922-1)

Between the pelvic elements described above, one very fragmentary and disarticulated sacrum was found. The centra are proportionally short, low and wide. Both proximal and distal articular surfaces are flat and very rough, indicating strong connections between adjacent centra. Between the articular surfaces, the centra are strongly constricted. On the dorsal side of the centra, the neural canal is very wide. The ventral side of the sacrum is neither grooved nor keeled. Sacral ribs were also found disarticulated between the pelvic elements.

## Discussion

### Phylogenetic Analysis

A phylogenetic analysis was conducted in order to assess the relationships of *Kundurosaurus nagornyi* within Saurolophinae. Although several phylogenies of saurolophines have recently been proposed [3], [22], [37], [38], our analysis is based on the data matrix published by Priéto-Márquez [20]. Indeed, this paper is the most comprehensive phylogenetic analysis of Hadrosauroidea to date. However, because of the large size of the original matrix (286 characters and 41 ingroup taxa), it is sometimes difficult to interpret the resulting cladogram. We have therefore decided to concentrate our own analysis on the saurolophines, because it is a priori clear that *Kundurosaurus nagornyi* is not a basal Hadrosauroidea or a Lambeosaurinae. We have also decided to exclude the OTUs that are not formally published yet and also *Shantungosaurus giganteus*, which clearly requires a systematic revision. Our data matrix is therefore limited to 21 OTUs. *Probactrosaurus gobiensis* and *Bactrosaurus johnsoni* have been chosen as successive outgroups, because they are fairly complete and familiar to the authors of the present paper. The number of characters considered in our analysis is consequently reduced too, because many of them became non-informative. We have also excluded several characters when we considered that their intraspecific variability was too high, that the preservation of the fossils could too easily influence the polarity of the character (this is particularly the case for characters based on angulations, which can easily be influenced by post-mortem crushing), or when the polarity was problematic (polarity unknown in outgroup taxa). The final matrix is consequently reduced to 176 characters. The character description (Text S1) and character-taxon matrix (Table S2) are presented as online supplementary information.

The 176 characters were equally weighted and analysed with TNT 1.1 [39]. A heuristic search of 10000 replicates using random addition sequences, followed by branch swapping by tree-bisection-reconnection (TBR; holding ten trees per replicate) was conducted. The trees were subsequently analysed using Winclada ver.1.00.08 [40] with fast and slow optimizations. To assess the repeatability of tree topologies, a bootstrap analysis was performed (1000 replicates with the heuristic algorithm in Winclada). Bremer support was assessed by computing decay indices with TNT 1.1.

**Figure 20:**
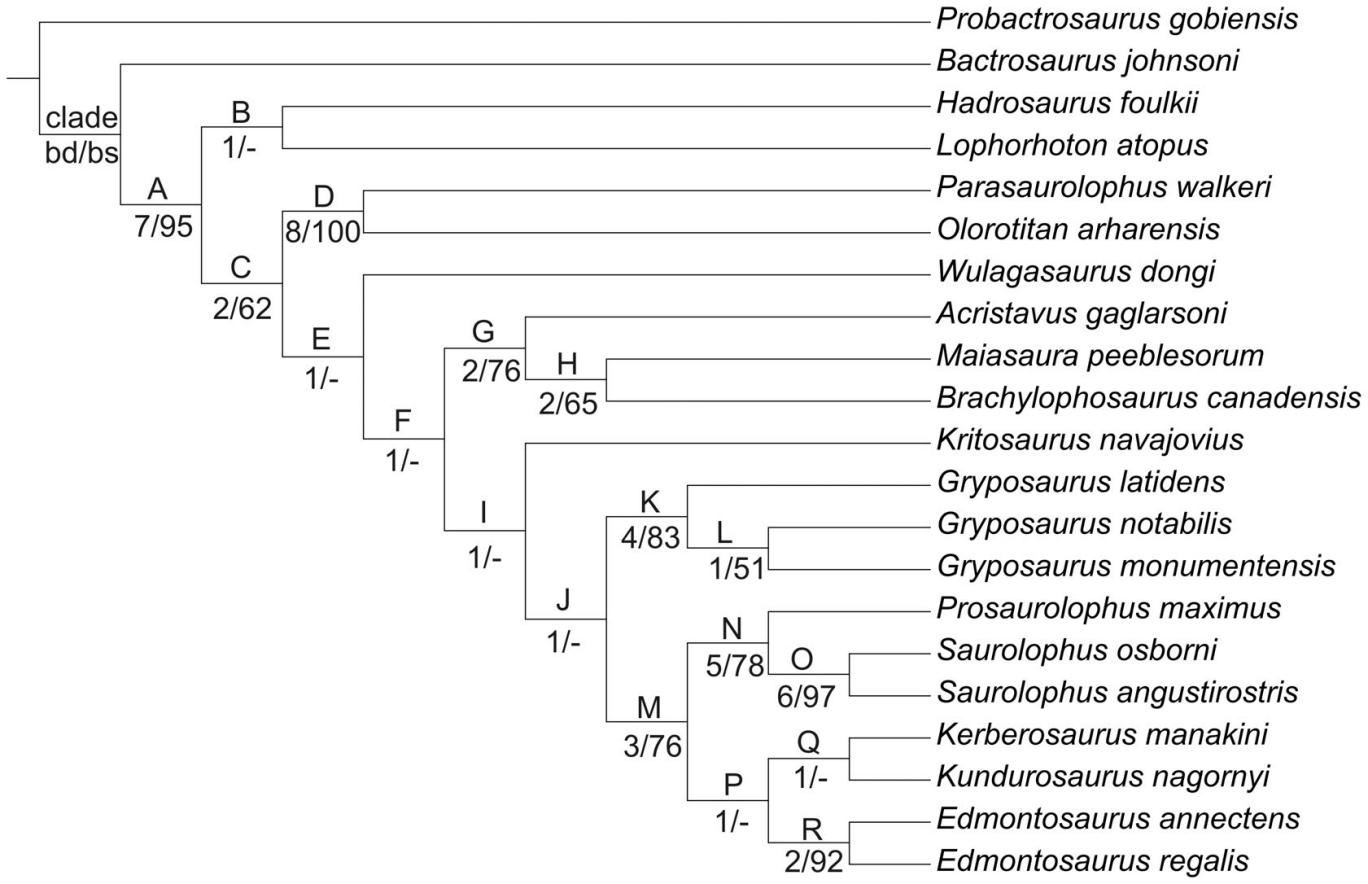
Phylogenetic analysis of Saurolophinae. Tree length  = 354 stps, CI = 0.68; RI = 0.75. Character list modified from [20], see Text S1 for the list of characters, Table S2 for the data matrix, and Text S2 for the tree description. **bd**, Bremer decay value; **bs**, bootstrap proportion. Bootstrap proportions lower than 50 are indicated by a hyphen.

The maximum parsimony analysis resulted in a single tree of 354 steps (Fig. 20). The consistency index (CI) is 0.68 and the retention index (RI) is 0.75. The tree description is presented as supplementary online information (Text S2). This analysis confirms that both *Lophorhothon atopus* and *Hadrosaurus foulkii* occupy a basal position, outside the clade Saurolophidae (defined as the last common ancestor of *Saurolophus osborni* and *Lambeosaurus lambei* and all of its descendants [20]). *Kundurosaurus nagornyi* is placed as the sister-taxon of *Kerberosaurus manakini*, also from the Maastrichtian of the Amur Region. It may therefore be postulated that *Kundurosaurus nagornyi* does not represent a separate genus, but is a second species of the genus *Kerberosaurus*. But this clade is particularly weakly supported and synapomorphies uniting both taxa can only been found under fast optimization. It means that the polarity of these characters is unknown in at least one of these two taxa and that it is currently impossible to propose a stable diagnosis of the genus *Kerberosaurus* including the species *manakini* and *nagornyi*. It reflects the fact that both taxa are represented by fragmentary specimens and that many characters usually regarded important from a phylogenetic point of view are lacking. However, significant differences can be observed on the few common elements:

Maxilla: the dorsal process appears rostrocaudally longer and more robust in *Kerberosaurus manakini*. Hook-like palatine process in *Kerberosaurus manakini*.
Nasal: more robust and more curved downwards in *Kundurosaurus nagornyi.* The crest that marks the dorsal and caudal limits of the circumnarial depression is much better developed in *Kundurosaurus nagornyi* (but it may be regarded as an ontogenetic character) and invades the caudal plate, whereas it closely follows the margin of the external naris in *Kerberosaurus manakini*. The caudal plate of the nasal is proportionally shorter in *Kundurosaurus nagornyi*.
Frontal: the frontals of *Kerberosaurus manakini* are particularly narrow and do not participate in the orbital margin.
Quadrate: much more robust and proportionally higer in *Kundurosaurus manakini*.
Parietal: its rostral margin is depressed around the contact area with the frontals in *Kerberosaurus manakini*.
Exoccipital: a strong ridge extends obliquely along the lateral side of the exoccipital condyloid in *Kundurosaurus nagornyi*.

Whether or not those difference are sufficient to merit generic distinction remains of course arbitrary. But in any case it is clear that those taxa must be treated as distinct operational taxonomic units in phylogenetic analyses. Because those taxa are clearly different and synapomorphies uniting them cannot be unambiguously defined, it as been decided to treat them as separate genera, pending the discovery of more complete material confirming or invalidating their generic distinction. It must also be noted that the four Maastrichtian dinosaur localities from the Zeya-Bureya Basin are also characterized by the presence of a distinct lambeosaurine genus (see below).

Whatever it may be, *Kundurosaurus nagornyi* and *Kerberosaurus manakini* are placed within the clade Edmontosaurini, characterized by three unambiguous (characters that do not change placement under both fast and slow optimizations) synapomorphies: supracranial crest absent (character 114 [0]), postacetabular process of ilium nearly as long as the central plate, ratio greater than 0.8 but less than 1.1 (character 154 [1], convergent in the brachylophosaurine clade), and proximal constriction of the prepubic process of the pubis longer than the dorsoventral expansion (character 160 [2]). However, this clade is also weakly supported (Bremer decay value = 1; bootstrap proportion <50). In this cladogram, the Edmontosaurini and Saurolophini clades form a rather well-supported (Bremer decay value = 3; bootstrap proportion = 76) monophyletic group, supported by the following unambiguous and unequivocal (CI = 1) synapomorphies: more than 42 tooth rows in the dentary dental battery (character 1 [2]), the medial or lateral profile of the dorsal margin of the rostral edentulous region of the dentary for articulation with the predentary has a very subtle concavity or is straight (character 23 [1]), margin of the dentary with a wide and well-developed ventral bulge rostral to the coronoid process (character 24 [1]), rostral end of the nasal at the contact with the dorsal process of the premaxilla long and subrectangular process, with slightly rounded corners (character 50 [2]), the nasal forms a greatly shortened and dorsoventrally narrow hook-like rostroventral process, exposed dorsal to the premaxillary caudoventral process (character 51 [2]), the triangular caudoventral expansion of the rostral process of the jugal forms a shallow and rostrocaudally wide prominence (wider than deep) (character 68 [1]), circumnarial fossa deeply incised (character 113 [1]) and sometimes invaginated in adults (character 113 [2]), and relatively long iliac peduncle of the ischium, ratio between the proximodistal length and the craniocaudal width of the distal margin greater than 2 (character 164 [1]). *Gryposaurus* is the sister-taxon of this Saurolophini + Edmontosaurini clade; however this monophyly is weakly supported by a single unambiguous and unequivocal synapomorphy: at least five teeth per alveoli arranged dorsoventrally at mid length of the dental battery (character 2 [2]).

Although they are basically based on the same data matrix, the cladogram of Saurolophinae presented here is clearly different from those published by Prieto-Márquez [20], more closely resembling the phylogenies previously published by Godefroit et al. [3], Bolotsky and Godefroit [5], and Bell [38]. The most important difference is the position of *Gryposaurus* and *Edmontosaurus.* According to Prieto-Márquez, *Edmontosaurus* is the sister-taxon of the monophyletic clade formed by Saurolophini + gryposaurs (including *Wulagasaurus dongi* and *Kritosaurus navajovius*). It is notable that both phylogenies are weakly supported, because only a few clades have a Bremer decay value greater than 1 and a bootstrap proportion greater than 50.

**Figure 21:**
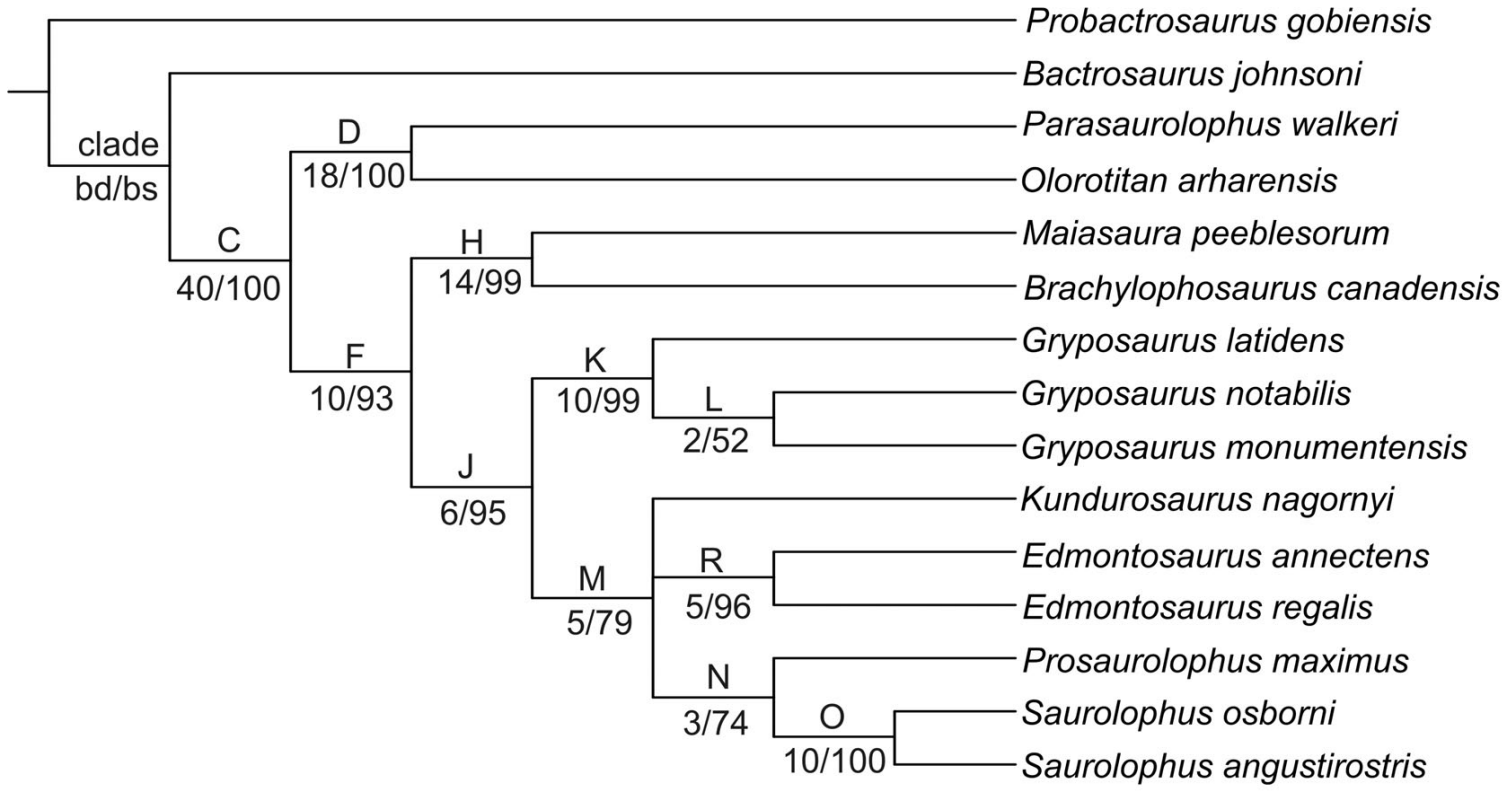
Simplified phylogenetic analysis of Saurolophinae. Strict consensus tree resulting from the parsimony analysis of 15 hadrosauroid taxa. Tree length = 315 stps, CI = 0.74; RI = 0.78. Character list modified from [20], see Text S1 for the list of characters, Table S2 for the data matrix, and Text S3 for the tree description. **bd**, Bremer decay value; **bs**, bootstrap proportion.

We have therefore decided to test the influence of missing data on the topology and robustness of the resulting cladogram and we have eliminated from the analysis taxa that are represented by too fragmentary specimens. However, we have kept *Kundurosaurus nagornyi*, keeping in mind that the ultimate aim of this analysis is to clarify its phylogenetic affinities within Saurolophinae. The maximum parsimony analysis resulted in two most parsimonious trees of 315 steps each with a consistency index of 0.74 and a retention index of 0.78. The consensus tree (Fig. 21; tree description in Text S3) shows that the general topology of the cladogram is kept (compare with Fig. 20), but that the robustness of the nodes is significantly increased. *Kundurosaurus nagornyi* is nested within an unresolved polytomy with *Edmontosaurus* and Saurolophini. This clade is rather robustly supported (Bremer decay value = 5, bootstrap proportion = 79). An additional analysis was constrained to produce a monophyletic group comprising *Gryposaurus* ssp. and Saurolophini, as hypothesized by Prieto-Márquez [20]. This analysis shows that this later hypothesis requires seven additional steps and is therefore less parsimonious.

### Paleogeography

So far, four main dinosaur localities are known along the borders of the Zeya-Bureya Basin. The distances between these localities are not important (see Fig. 1) and the saurolophid fossils have been discovered in the same *Wodehouseia spinata*- *Aquilapollenites subtilis* palynozone, suggesting that these hadrosaurs are roughly synchronous, from a geological point of view. *Kundurosaurus nagornyi* is the third saurolophine discovered in the Zeya-Bureya Basin. *Kerberosaurus manakini* is known from disarticulated skull material from the Udurchukan Formation at Blagoveschensk [7] and *Wulagasaurus dongi*, from disarticulated bones from the co-eval Yuliangze Formation at Wulaga in China [3]. *Mandschurosaurus amurensis* and *Saurolophus kryschtofovici*, both from the Yuliangze Formation at Jiayin (China) are now unanimously regarded as *nomina dubia* [41]. Although the holotype specimen of *Mandschurosaurus amurensis* is clearly a chimera, reconstructed from several individuals, several of its bones (humerus, part of the mandible) apparently belong to saurolophines. A partial left dentary with dozens of teeth from Jiayin [42] clearly belongs to a saurolophine and probably to ‘Node J’ in Figures 20–21, like *Kundurosaurus nagornyi* and *Kerberosaurus manakini*. Indeed, at least five teeth per alveoli are dorsoventrally arranged at mid length of the dental battery (character 2 [2]), which is an unambiguous and unequivocal synapomorphy for this clade. The dentary crowns of this specimen are characterized by the presence of well-developed secondary and tertiary ridges, an unusual character in saurolophines. Dentary teeth are unfortunately not associated with *Kundurosaurus nagornyi* dentaries, so it is not possible to know whether the saurolophine dentary from Jiayin belongs or not to the new taxon. The same apparent patchy distribution can also be observed in lambeosaurine saurolophids from the Amur region: *Charonosaurus jiayinensis* is limited to Jiayin locality, *Sahaliyania elunchunorum* to Wulaga, *Amurosaurus riabinini* to Blagoveschensk, and *Olorotitan arharensis* to Kundur locality. Ecological factors, which still have to be investigated, therefore probably lead to an important habitat partitioning of hadrosaurid faunas in eastern Asia during the Maastrichtian. Similar habitat partitioning has also been observed in North American hadrosaurids [37]. Important habitat partitioning between species that have a great potential for dispersion suggests that competition for food resources was very important between hadrosaurid populations that lived in the Amur-Heilongjiang region at the end of the Cretaceous. In modern large vertebrates, important habitat partitioning usually implies an elaborated social live. It has been postulated that hadrosaurid circumnasal and supracranial features may have been used for both visual and vocal communication, and were implied in species recognition, intraspecific combat, ritualised display, courtship display, parent-offspring communication and social ranking. They would have promoted successful matings within species that live close from each other by acting as premating genetic isolating mechanisms [37], [43].

Figure 20 suggests that *Kundurosaurus nagornyi* and *Kerberosaurus manakini* belong to a single clade and that their presence in Maastrichtian deposits from Far Eastern Russia may be explained by the local evolution of a single saurolophine lineage. However, *Wulagasaurus dongi* is here regarded as the most basal Saurolophine (*contra* [20]). If this interpretation is correct, its presence in Maastrichtian deposits from Eastern Asia implies a long ghost lineage for basal saurolophines in Asia. Lambeosaurines from the Amur region also belong to well separated lineages: *Amurosaurus riabinini* is a basal lambeosaurine [4], *Sahaliyania elunchunorum* is a more advanced lambeosaurine [3], *Charonosaurus jiayinensis* is regarded as the sister-taxon of the North-American genus *Parasaurolophus* [41], and *Olorotitan arharensis* belongs to the same clade as the North-American genera *Hypacrosaurus* and *Corythosaurus* [44]. Such a diversity and mosaic distribution of Maastrichtian saurolophid faunas in the Amur-Heilongjiang region is the result of a complex paleogeographical history and implies that many independent hadrosaurid lineages dispersed without any problem between western America and eastern Asia at the end of the Cretaceous. Fiorillo [45] recently demonstrated that the concept of Beringia, an entity encompassing northeastern Asia, northwestern North America and the surmised land connection between the two regions, should be formally extended back in time to the Cretaceous and is rooted in its accretionary rather than its climatic history. Godefroit et al. [46] showed that the Late Maastrichtian Kakanaut dinosaur fauna in Chukotka (northeastern Russia) more closely resembles the Hell Creek fauna of western North America than the synchronous Amur-Heilongjiang fauna. All this partial data suggest that the evolutionary history and paleogeography of dinosaur faunas in eastern Asia is still very partially understood. The huge territories of Far Eastern Russia, which have been poorly explored so far, have a great potential for new discoveries that would bring clues to clarify this complex situation.

### Nomenclatural Acts

The electronic version of this document does not represent a published work according to the International Code of Zoological Nomenclature (ICZN), and hence the nomenclatural acts contained in the electronic version are not available under that Code from the electronic edition. Therefore, a separate edition of this document was produced by a method that assures numerous identical and durable copies, and those copies were simultaneously obtainable (from the publication date noted on the first page of this article) for the purpose of providing a public and permanent scientific record, in accordance with Article 8.1 of the Code. The separate print-only edition is available on request from PLoS by sending a request to PLoS ONE, 185 Berry Street, Suite 3100, San Francisco, CA 94107, USA along with a check for $10 (to cover printing and postage) payable to “Public Library of Science”. In addition, this published work and the nomenclatural acts it contains have been registered in ZooBank, the proposed online registration system for the ICZN. The ZooBank LSIDs (Life Science Identifiers) can be resolved and the associated information viewed through any standard web browser by appending the LSID to the prefix “http://zoobank.org/”. The LSID for this publication is: urn:lsid:zoobank.org:pub: 9109412E-8B2F-4010-9828-63C7D2BC7340.

## Supporting Information

Table S1 **Measurements.**
(DOCX)

Table S2 **Character-taxon matrix.**
(DOCX)

Text S1 **List of characters used in the phylogenetic analysis.**
(DOCX)

Text S2 **Tree description of the larger cladogram (** **Fig. 20** **).**
(DOCX)

Text S3 **Tree description of the reduced cladogram (** **Fig. 21** **).**
(DOCX)
