# Supplementary material for: A New Saurolophine Dinosaur from the Latest Cretaceous of Far Eastern Russia
Source: PLoS One. 2012 May 30;7(5):e36849. doi: 10.1371/journal.pone.0036849 (PMC3364265; doi:10.1371/journal.pone.0036849)
Supplement: Table S1 — Measurements. (DOCX) [file pone.0036849.s001.docx]

**Table S1: Measurements**

Jugal (AENM 2/921), left

Length: 325 mm

Height of rostral process: 136 mm

Minimum depth of rostral constriction: 66 mm

Dorsoventral depth of the flange: 101 mm

Minimum depth of the caudal constriction: 77.5 mm

Distance between the point of maximum curvature of the infratemporal margin and the caudal margin of the lacrimal process: 125 mm

Quadrate (AENM 2/723), right

Height: 408 mm

Distance from mid-height of quadrate notch to quadrate head: 285 mm

Length of distal surface: 57 mm

Width of distal surface: 64.5 mm

Frontal (AENM 2/723), left

Length: 172 mm

Maximal width: 110 mm

Sternal (AENM 2/913), right

Total length: 445 mm

Length of craniomedial plate: 180 mm

Length of caudoventral process: 300 mm

Scapula (AENM 2/913), right

Dorsoventral depth of the proximal region: 109 mm

Dorsoventral depth of the distal end: + /- 95 mm

Dorsoventral depth of proximal constriction: +/- 77 mm

Distance from the coracoid joint and the cranial end of the acromion process: 76 mm

Height between the cranial end of the acromion process and the ventral apex of the glenoidal facet: 83 mm

Humerus (AENM 2/203), right

Length: 303 mm

Length of deltopectoral crest: 156 mm

Width of deltopectoral crest: 71 mm

Width of distal shaft at the point of maximum curvature: 41 mm

Width of the lateral surface of the proximal end: 78 mm

Ulna (AENM 2/905), right

Length: 710 mm

Mesiolateral width of proximal end: 138 mm

Dorsoventral thickness (measured at mid-shaft): 61 mm

Radius (AENM 2/904), right

Length: 607 mm

Mesiolateral width of proximal end: 79 mm

Dorsoventral thickness (measured at mid-shaft): 51 mm

Ilium (AENM 2/922), right

Length: +/- 930 mm

Length of preacetabular process: +/- 400 mm

Dorsoventral distance between the pubic peduncle and the dorsal margin of the ilium: 193 mm

Depth of the proximal region of the preacetabular process: 117 mm

Distance between the pubic peduncle and the caudodorsal prominence of the ischial peduncle: 275 mm

Length of the central plate: 283 mm

Depth of the central plate: 187 mm

Length of postacetabular process: 265 mm

Pubis (AENM 2/922), left

Craniocaudal length of the pubis (distance from the acetabular margin to the distal margin of the prepubic process): +/ - 530 mm

Distance from the dorsal margin of the iliac peduncle to the ventral margin of the proximal postpubic shaft: 153.5 mm

Width of the acetabular margin of the pubis: 154 mm

Depth of the dorsoventral expansion of the distal region of the prepubic

process: +/- 184 mm

Length of proximal constrition of prepubic process: 310 mm

Length of the dorsoventral expansion of the prepubic process: +/- 220 mm

Length of ischial peduncle: 74.5 mm

Width of ischial peduncle: 32 mm

Length of iliac peduncle: 87 mm

Ischium (AENM 2/922), left

Length : 792 mm

Length of iliac peduncle: +/- 140 mm

Width of iliac peduncle: 66 mm

Length of pubic peduncle: 38 mm

Width of pubic peduncle: 77 mm

Length of the ischial shaft: 590 mm

Dorsoventral thickness of the midshaft: 29 mm
