# Supplementary material for: A New Saurolophine Dinosaur from the Latest Cretaceous of Far Eastern Russia
Source: PLoS One. 2012 May 30;7(5):e36849. doi: 10.1371/journal.pone.0036849 (PMC3364265; doi:10.1371/journal.pone.0036849)
Supplement: Table S2 — Character-taxon matrix. (DOCX) [file pone.0036849.s002.docx]

**Table S2: character-taxon matrix**

*Probactrosaurus gobiensis* ?00000000??0000000?0?000000000000?00??000000000000???000000????00000?00??0???????0?????000?00000(01)(01)??????0000?0???0?0???0???00000000000000000000000000000000000000000000000000000

*Bactrosaurus johnsoni* 0000000200000010000000000000000010100?1001000000000?00000100000010000010000000000000000000000000000000000010?0000000000100000010000001000101000?12001011100000?00000110000110010

*Parasaurolophus walkeri*

111???????111?11310?01001101111111110?11110000/1?////101211?01122130100212111001121000121111111101????10111/12//6111210?101101011111110002211011201312120111112101011100011????

*Olorotitan arharensis*

11111102111?1111211101001101111111110?111100001/1?////1012112?1122130100112?11001101000111011111101100010?11/12//611?21001011011111111100022111?1201112121(01)11?????1?11100011????

*Maiasaura peeblesorum* 1112110211?21112111111001111111111111?213110100001110001021111112012011110101001100110102110002011111101111000120411100111001111111111110110101112012031211111211000111101110011

*Brachylophosaurus canadensis* 1112110211121112111201001111111111111?21311010000111000102111111201201111(01)10?000100110101111002011111101111010130211100111001111111111110110101112012031211111211000111101110011

*Acritavus gaglarsoni* 1112110?111????????21100110111111???????311010000???000102111?1120120111101??010001??00021100000011111?11110?01????110?1????????????????????????????????????????1?????????11????

*Gryposaurus notabilis* 1211101211?00010310201001101111111111?112110100100?02202021110112112110210001?100001110011000020011?000121103011011101000110111111110011111112111111212122?111411100111111111110

*Gryposaurus monumentensis* 1212101211101010310201001101111111111?11211010010010220202111011211211121100101000011100?100002001?1?001?110301101110100????????????????????????????????????????????????????????

*Gryposaurus latidens* 1212121111??????3??201001101111111111?21211??00?0010?102021110112????????????0?0??0???00???0??????????????10?011011?0???011?1111111100111111120?11?121212211114111???????1????10

*Kritosaurus navajovius* 111110?221?0?11011?001?01101111111111???????????001?100102111?1121121112101010100001110001?00000111100012110?01205110100?1??????????????????????????????????????????????????????

*Prosaurolophus maximus* 2212101221?01012311101111101111111111?2111101000022110010211101?21111102010110111011100011?0102011110?012110201225111000?1101?11111111111?111(0 2)111211(2 3)121221111411101111101110110

*Saurolophus osborni* 2212101221?01?10311201111101111111111?1111101000022100010211101121111102011110011121100021?01021?11100012110201323111000111111111111111111?211011211312122111141100?1???01111110

*Saurolophus angustirostris* 221210?221?01010311201111101111111111?111110100002210001021110112111110201111001112110002100102011110001211020132311100011111111111111111122110112113121221111411001111001111110

*Edmontosaurus annectens* 2212100221111?11311301111101111111111?213111110002211001021111112111110221111010001200001100002021110011211010112011100101101111111111110121101112112121211111321101111111110010

*Edmontosaurus regalis* 2212100221111?11311301111101111111111?213111110002211001021111112111110221111010001200001100002021110011?11010112011100101101111111111110121101112012121211111321001111111111010

*Hadrosaurus foulkii* ???21011????????????0???????????????????????????????????????????2?????????????????????????????????????????????????????????????????????????00001?12012??12??10??????01100?111????

*Lophorhoton atopus* 10?110010???????????????????????????????????????0???0001????????200??0?????0100000?0??000100000??0????0?01?0?0?1?5???000?????????????????????????????????????????????????11?0000

*Wulagasaurus dongi* 1??????????????????1???0110111111????????????????????????2111?11201??1????1?1??????????????????0?0????01101????????????????????????11??1?11210???????????????????????1??11??????

*Kerberosaurus manakini* ????????21??????????????????????????????????????0????00102111??121111102111?10100??????0?1001020?111????2110?01110??????????????????????????????????????????????????????????????

*Kundurosaurus nagornyi*  2??????????????????????1110111111???????????????02??20??021????1211111021111???0?1?1??00??000020?111?00?2110?01110?1?????????1?????110?11?11111?0211(1 2)121211111221101111111??????
