# Supplementary material for: A New Saurolophine Dinosaur from the Latest Cretaceous of Far Eastern Russia
Source: PLoS One. 2012 May 30;7(5):e36849. doi: 10.1371/journal.pone.0036849 (PMC3364265; doi:10.1371/journal.pone.0036849)
Supplement: Text S1 — List of characters used in the phylogenetic analysis. (DOCX) [file pone.0036849.s003.docx]

**Text S1: List of characters used in the phylogenetic analysis (based on [20])**

Dental characters:

1. Maximum number of tooth positions in the dentary dental battery: 30 or less (0); 31–42 (1); more than 42 (2). ([20], character 1). Character treated as ordered.
2. Minimum number of teeth per alveoli arranged dorsoventrally at mid length of the dental battery: maximum three (0); four (1); five (2). ([20], character 2, modified).
3. Maximum number of functional teeth exposed on the dentary occlusal plane: one or two (0); three functional teeth throughout most of the dental battery, gradually decreasing to two near the rostral and caudal ends of the dentary (1). ([20], character 2, modified).
4. Maximum number of ridges on the enamelled lingual side of dentary tooth crowns: presence of primary, secondary, and one or two tertiary ridges (0); presence of a primary ridge and one or two faint and shorter ridges (1); loss of all but primary ridge (2). ([20], character 5, modified). Character treated as ordered.
5. Dentary tooth crowns, position of primary ridge: well offset caudally from the midline (0); median for most teeth, although some teeth within the same dental battery may display a slight caudal offset of the primary ridge (1). ([20], character 6).
6. Shape of the primary ridge of dentary tooth crowns: straight in all teeth within the same dentition (0); straight for some crowns and sinuous for others. ([20], character 7).
7. Angle between the crown and the root of dentary teeth: more than 135° (0); up to 135° (1). ([20], character 8, modified).
8. Overall morphology of dentary marginal denticles: wedge to tongue-shaped (0); curved and mammillated asymmetrical ledge (1); absent or very reduced to small papillae along the apical half of the crown (2). ([20], character 9).
9. Maximum number of tooth positions in the maxillary dental battery: up to 32 tooth positions (0); from 33 to 44 tooth positions (1); 45 or more tooth positions (2). ([20], character 15). Character treated as ordered.
10. Maximum number of functional teeth per alveolus in the maxillary occlusal plane: one tooth for most of the dental batery, with the sporadic presence of a second tooth forming the occlusal plane (0); two functional teeth throughout most of dental battery length, gradually changing to one near the rostral and caudal ends of the maxilla (1). ([20], character 16, modified).
11. Overall morphology of the maxillary marginal denticles: curved and mammillated asymmetrical ledge (0); absent or reduced to small papillae along the apical half of the dorsal half of the crown (1). ([20], character 21, modified).

Predentary

1. Ratio between the predentary maximum mediolateral width and the maximum rostrocaudal length along the lateral process: less than 1.2 (0); between 1.2 and 1.75 (1); more than 1.75 (2). ([20], character 22).
2. Shape of the denticles of the predentary oral margin: triangular and pointed (0); subtriangular to subrectangular (1). ([20], character 25, modified).
3. Number of predentary denticles in adults lateral to the median denticle (not included in the count): maximum of five (0); six or more (1). ([20], character 27, modified).
4. Extension of predentary denticulate margin: denticles extending on lateral process (0); denticles limited to the rostral margin (1). ([20], character 28).
5. Morphology of predentary rostrolateral corner: gently rounded and continuous with the lateral process, giving the predentary an arcuate dorsal profile (0); subsquared rostrolateral corner (1); subsquared, very broad, and rostrolaterally projected (2). ([20], character 29).
6. Development of a lateral shelf on the dorsal side of the predentary lateral process: short and shallow shelf, limited to the laterocaudal region of the lateral process (0); short and well-incised shelf that is wider near the rostrolateral corner of the predentary (1); shelf extremely narrow mediolaterally and very long rostrocaudally (2); shelf rostrocaudally long, deeply incised and mediolaterally broad, forming half of the mediolateral breadth of the lateral process and becoming wider distally (3). ([20], character 30, modified).
7. Ridge on the dorsal lingual, keel-like process of the predentary: the process lacks a prominent median ridge on the lingual side of the rostral region of the predentary, and, if present, the former forms and projects caudally from the caudal margin of the predentary rostral region (0); the process has a well-developed ridge on the lingual surface of the rostral segment of the predentary, from which the former extends further caudally to lie dorsal to the dentary symphysis (1). ([20], character 31).
8. Degree of indentation of the split of the predentary ventral median processes into two distinct lobes: short indentation and deep undivided portion (0); long indentation and shallow undivided portion (1). ([20], character 32).

Dentary

1. Ratio between the length of the proximal edentulous slope of the dentary and the distance between the rostralmost tooth position and the caudal margin of the coronoid process: less than 0.20 (0); ratio between 0.20 and 0.31 (1); ratio between 0.32 and 0.45 (2); ratio greater than 0.45 (3). ([20], character 33).
2. Lingual projection of the symphyseal region of the dentary (measured as a ratio between the labiolingual extension of the symphyseal region and the maximum labiolingual width of the dentary): ratio greater than 1.65 and up to 2.85 (0); ratio up to 1.65 (1). ([20], character 38, modified).
3. Orientation of the dentary symphysis (measured as the angle formed by this surface and the lateral side of the rostral half of the dentary): angle greater than 15°; angle up to 15° (1). ([20], character 39).
4. Medial or lateral profile of the dorsal margin of the rostral edentulous region of the dentary for articulation with the predentary: having a well-pronounced concavity (0); ranging from having a very subtle concavity to straight (1). ([20], character 40, modified).
5. Bulging of the ventral margin of the dentary: margin straight or slightly bowed rostral to coronoid process (0); margin with a wide and well-developed ventral bulge rostral to the coronoid process (1). ([20], character 41, modified).
6. Orientation of coronoid process: subvertical or caudally inclined(0); rostrally inclined (1). ([20], character 42, modified).
7. Morphology of the apex of the coronoid process: slightly expanded rostrocaudally, with very limited development of rostral and caudal expansions resulting in an apex that is taller than wider (0); well-developed expansion of both the caudal and, especially, the rostral margins (1). ([20], character 43).
8. Caudodorsal margin of the coronoid process projected dorsally into a sharp point: absent (0); present (1). ([20], character 43).
9. Thick and dorsoventrally elongated ridge on the medial side of the coronoid process: absent, presence of fine striations (0); present, the ridge forms the rostral boundary of a depressed facet for attachment of the rostrodorsal process of the surangular, coarse striations present rostral to the ridge (1). ([20], character 45).
10. Lateral expansion of the caudal region of the dentary, ventral to the base of the coronoid process (measured as the angle between the lateral surface of the dentary and that of the region caudoventral to the coronoid process): the lateral side of the dentary is only slightly expanded laterally ventral to the coronoid process, with an angle greater than 165° (0); well-developed expansion of the lateral side of the dentary ventral to the coronoid process, with an angle of up to 165° (1). ([20], character 46).
11. Orientation of the longitudinal axis of the dentary occlusal plane relative to the lateral side of the bone: diagonal axis, directed rostrolaterally and forming approximately 15° with the lateral side of the dentary (0); axis parallel to the lateral side of the dentary (1). ([20], character 47).
12. Lingual arching of the occlusal plane: present, lingually convex occlusal plane (0); absent, rostrocaudally straight occlusal plane (1). ([20], character 48).
13. Caudal extension of the dental battery: flush with the caudal margin of the coronoid process (0); caudal to the caudal margin of the coronoid process (1). ([20], character 49, modified).
14. Separation between the dentary tooth row and the coronoid process: the coronoid process is laterally offset (but nearly in contact) with the tooth row, lacking a platform in between the tooth row and the base of the process (0); the coronoid process is laterally offset relative to the tooth row, with the presence of a concave platform or, in some cases, a laterodorsal concave slope separating the base of the process from the dental battery (1). ([20], character 50).

Surangular

1. Morphology of the rostrodorsal process of the surangular: rostrocaudally thick process extensively exposed in lateral view (0); rostrocaudally reduced in thickness, strap-like and wedging dorsally into a thin sliver that becomes concealed in lateral view by the dorsal half of the caudal margin of the coronoid process (1). ([20], character 51).
2. Surangular foramen: present (0); absent (1). ([20], character 52).
3. Orientation of the convex side of the lateral lap and the lateroventral surface of the main body of the surangular: facing more laterally than ventrally (0); facing more ventrally than laterally (1). ([20], character 52).
4. Lateral curvature of the caudal process of the surangular: present, process laterally recurved (0); absent, process nearly straight rostrocaudally (1). ([20], character 55, modified).

Angular

1. Position of the angular in the mandible: positioned ventrally and slightly medially, exposed in lateral view (0); positioned medially, not exposed in lateral view (1). ([20], character 57).

Premaxilla

1. Mediolateral expansion of the premaxillary oral margin (measured as the ratio between the maximum mediolateral width of the premaxilla and the minimum width at the narrowest point or post-oral constriction): relatively narrow, ratio less than 1.65 (0); ratio between 1.65 and 2 (1); very wide, with a ratio greater than 2 (2). ([20], character 60). Character treated as ordered.
2. Position of the premaxillary oral margin relative to the occlusal plane of the dentition: premaxillary margin slightly ventrally offset from occlusal plane (approximately, the dorsoventral distance between the occlusal plane and the level of the premaxillary oral margin is less than the mean depth of the dentary) (0); very strongly deflected ventrally (approximately, the dorsoventral distance between the occlusal plane and the level of the premaxillary oral margin is equal to or larger than the mean depth of the dentary) (1). ([20], character 61).
3. Degree of expansion and folding of the oral margin of the premaxilla: moderately expanded border, becoming thinner towards the parasagittal plane of the snout (0); folded caudodorsally into a thin recurved margin (1); ventrally deflected and dorsoventrally expanded, forming a very thick 'lip-like' margin (2). ([20], character 62, modified).
4. Premaxillary oral margin with a double layer morphology consisting of an external denticle-bearing layer and an internal layer of thickened bone, set back slightly from the oral margin, and separated from the denticular layer by a deep sulcus bearing vascular foramina: absent (0); present (1). ([20], character 63).
5. Premaxillary foramen located rostrally and ventrolaterally to the rostral margin of the external naris: absent (0); present (1). ([20], character 64).
6. Premaxillary accessory foramen entering rostrally through the outer (rostral) narial fossa, located rostral to the premaxillary foramen: absent (0); present, empties into a common chamber with the premaxillary foramen (1). ([20], character 65).
7. Premaxillary accessory narial fossa located rostral to the circumnarial depression: absent (0); present, separated from circumnarial depression by a rostrocaudally wide ridge (1). ([20], character 66).
8. Premaxillary additional accessory fossa located lateral to the rostral accessory fossa and rostrolateral to the circumnarial depression, parallel with the lateral border of the oral margin: absent (0); present (1). ([20], character 67).
9. Elongation of premaxillary caudodorsal process: the premaxillary caudodorsal process does not meet the caudoventral process caudally (0); elongate caudodorsal process that extends caudally to meet the caudoventral process, forming the caudal margin of the external naris (1). ([20], character 68).
10. Dorsolateral flange at approximately mid-length of the caudoventral process of the premaxilla: absent (0); present (1). ([20], character 74).

Nasal

1. Location of the nasal bone and nasal cavity in the adult skull: the nasal extends from the rostral region of the skull to the rostrodorsal region of the snout with the nasal cavity rostromedial to the orbit (0); nasal retracted caudal to the rostrum, resulting in a supracranial hollow crest (1). ([20], character 75, modified).
2. Morphology of the rostral end of the nasal at the contact with the dorsal process of the premaxilla: long and wedge-shaped rostral process, gradually decreasing in width rostrally to a sharp point (0); hook-like process, it becomes abruptly deep near the rostral end and then wedges rostrally (1); long and subrectangular process, with slightly rounded corners (2). ([20], character 77, modified).
3. Morphology of the nasal contact with the caudodorsal region of the caudoventral premaxillary process at the caudal margin of the narial foramen: the nasal forms a subrectangular flange exposed dorsal to the premaxillary caudoventral process (0); the nasal forms a large hook-like rostroventral process, exposed dorsal to the premaxillary caudoventral process (1); the nasal forms a greatly shortened and dorsoventrally narrow hook-like rostroventral process, exposed dorsal to the premaxillary caudoventral process (2). ([20], character 78).
4. Location of the rostral end of the dorsal process of the nasal relative to the rostral margin of the external naris: the rostral end of the rostrodorsal process of the nasal does not reach the rostral margin of the narial foramen (0); the rostral end of the rostrodorsal process of the nasal reaches the rostral margin of the narial foramen (1). ([20], character 79).
5. Caudal processes of the nasals: absent (0); forming a pair of finger-like processes on top of the frontals and centered around the sagittal plane of the skull roof (1); forming a pair of small and short processes that insert between the frontals at the sagittal plane of the skull roof (2). ([20], characters 81-82, modified).
6. Nasal arch: absent, dorsal border of the rostral process of nasal at about the same level as the caudal plate (0); present, summit located dorsal to the caudal margin of the narial foramen (1); present, summit located caudodorsal to the caudal margin of the narial foramen (2). ([20], characters 83, modified).

Maxilla

1. Rostrodorsal process that is medially offset from the body of the maxilla, and also extends medial to the caudovenventral process of the premaxilla to form part of the medial floor of the external naris: present (0); absent, the rostral end of the maxilla forms a ventrally sloping rostrodorsal shelf that underlies the premaxilla (1). ([20], character 84).
2. Position of the base of the dorsal process: base of dorsal process positioned caudal to the mid-length of the maxilla (0); base of dorsal process centered around the mid-length of the maxilla (1); base of dorsal process located rostral to the mid-length of the maxilla (2). ([20], characters 90, modified).
3. Morphology of the apex of the dorsal process of the maxilla: subtriangular, not dorsoventrally taller than rostrocaudally wide (0); dorsoventrally taller than it is wide, with a peaked and caudally inclined apex (1). ([20], character 91).
4. Morphology of the jugal articulation surface: finger-like process (0); dorsolaterally-facing joint surface for the jugal with a caudolaterally directed corner (1); laterally-facing joint surface with a lateroventrally-directed pointed corner (2). ([20], characters 92, modified).
5. Arrangement of maxillary foramina ventral and rostral to the jugal articulation (excluding large rostrodorsal or rostrolateral foramen): positioned rostrocaudally and scattered throughout the lateral side of the maxilla (0); forming either a row or cluster that is oriented rostrodorsally (1). ([20], character 93).
6. Number of maxillary foramina ventral and rostral to the jugal articulation (excluding large rostrodorsal or rostrolateral foramen): seven or more (0); six or less (1). ([20], character 94).
7. Large rostral maxillary foramen: opening on the rostrolateral body of the maxilla, within the rostral half of the rostrodorsal margin of the element, and exposed in lateral view (0); opening on the rostrolateral body of the maxilla, within the dorsal half of the rostrodorsal margin of the element, and exposed in lateral view (1); opening on the dorsal surface of the maxilla along the maxilla-premaxilla contact, not exposed laterally (2). ([20], character 95).
8. Maxilla-lacrimal contact: present externally (0); largely covered externally by the jugal-premaxilla contact (1). ([20], character 96).
9. Length of the ectopterygoid shelf relative to the total rostrocaudal length of the alveolar margin of the maxilla: ratio greater than 0.25 and up to 0.35 (0); ratio greater than 0.35 (1). ([20], character 97, modified).
10. Slope of the ectopterygoid shelf, measured as angle between this and the rostrocaudal axis of the caudal portion of the tooth row: steeply inclined caudoventrally, with an angle greater than 21° (0); slightly inclined (angle less than 20°) or nearly horizontal (1). ([20], character 98, modified).
11. Morphology of the lateral emargination of the ectopterygoid shelf: dorsoventrally thin ridge (0); faint or dorsoventrally thin rostrally, then abruptly becoming dorsoventrally thick along the caudal segment of the margin (1); dorsoventrally thick continuous ridge, gradually thicker caudally than rostrally (2). ([20], character 99).

Jugal

1. Rostral apex of the rostral process: present, wedge-shaped, elongated and sharply pointed, positioned at mid-distance along the dorsoventral depth of the rostral process (0); present, wedge-shaped, pointed and less elongated than in (0), positioned within the dorsal half of the rostral process of the jugal; the dorsal magin of the apex forms a steeper angle with the horizontal than in state (0) (1); reduced to a blunt convexity or straight (2). ([20], character 103, modified).
2. Dorsoventral expansion of the caudodorsal margin of the rostral process: dorsoventrally narrow, rostrodorsally directed and forming little of the rostroventral margin of the orbital rim (0); dorsoventrally deep (about 60-90% as deep as the rostral jugal constriction), dorsally or slightly recurved caudodorsally, forming the rostroventral corner of the orbital rim (1). ([20], character 104).
3. Morphology of the triangular caudoventral expansion of the rostral process of the jugal: no expansion (0); shallow and rostrocaudally wide prominence (wider than deep) (1); ventrally pointed, approximately as deep as or slightly deeper as its proximal end is wide (2); ventrally projected triangular narrow process, at least twice as deep as it is wide, sharply pointed and often recurved caudally (3). ([20], character 105, modified).
4. Location of the caudoventral apex of the rostral process relative to the caudodorsal articulation with the lacrimal (with longitudinal axis of the rostral process oriented horizontally): apex located ventral to the caudal margin of the lacrimal process (0); apex located ventral to the caudal margin of the lacrimal process (1). ([20], character 106).
5. Orientation of the medial articular surface of the rostral process of the jugal: facing medioventrally, the articular surface forms a deep concavity bounded dorsally and caudally by a laterally offset rim (0); facing medially, the articular surface is bounded only caudally by a rim of bone (1). ([20], character 107).
6. Ventral expansion of the caudoventral jugal flange (measured as the ratio between the dorsoventral depth of the flange and the minimum depth of the caudal constriction of the jugal): slightly expanded flange, ratio of 1.55 or less (0); greatly expanded flange, ratio greater than 1.55 (1). ([20], character 110, modified).
7. Lateral profile of the quadratojugal flange: auricular in shape, with subparallel concave to nearly straight dorsal and convex ventral margins that converge dorsally into a short, subconical point (0); fanlike, with dorsal and ventral margins that are subparallel and diverge caudodorsally, dorsal and ventral margins can be straight or slightly bowed dorsally (1); auricular in shape, with subparallel concave to nearly straight dorsal and convex ventral margins that converge dorsally into a recurved or dorsally-directed tall subconical extension [this state is similar to (1), but the dorsal region of the flange is rostrocaudally narrower and taller] (2). ([20], character 111, modified).
8. Relative depth of the caudal and rostral constrictions (in adults) (rostral constriction: region located between the rostral and postorbital processes; caudal constriction: region located between the postorbital process and the caudoventral flange): deeper rostral constriction, ratio of the depth of the caudal constriction relative to the rostral of 1 or less (0); deeper caudal constriction, with a ratio greater than 1 and less than 1.35 (1); much deeper caudal constriction, with a ratio greater than 1.35 (2). ([20], character 113).
9. Jugal overall robustness (in adults), measured as the ratio between the minimum depth of the caudal constriction and distance between the point of maximum curvature of the infratemporal margin and the caudal margin of the lacrimal process: relatively gracile jugal, ratio less than 0.60 (0); relatively robust jugal, ratio of 0.60 or greater (1). ([20], character 114).
10. Relative width and lateral profiles of the orbital and infratemporal margins of the jugal: wider infratemporal margin (0); orbital and infratemporal margins are nearly equally wide (1); wider orbital margin (2). ([20], character 115, modified).

Quadrate

1. Development of the squamosal buttress on the caudal margin of the dorsal end of the quadrate: present, the squamosal buttress is a sharp protuberance hanging from the caudal side of the dorsal fourth of the quadrate, near the head of the element (0); absent or poorly developed as a gentle convexity (1). ([20], character 120, modified).
2. Morphology of the ventral surface of the quadrate: mediolaterally broad and rostrocaudally compressed, lateral condyle slightly larger than the medial one; the ventral surface of the lateral condyle is only slightly offset ventrally relative to the ventral surface of the medial condyle (0); subtriangular in ventral view, lateral condyle rostrocaudally expanded and much larger than the medial one; the ventral surface of the lateral condyle is well offset ventrally relative to the ventral surface of the medial condyle (1). ([20], character 121).

Prefrontal

1. Dorsomedial margin of the prefrontal developed into a caudodorsally-oriented crest: absent (0); present (1). ([20], character 122).
2. Lateral profile of the rostrodorsal margin of the prefrontal: subarcuate to smoothly curved, the rostral margin is rostroventrally oriented and forming an obtuse angle with the dorsal orbital margin (0); rostromedially broad with subsquared rostrodorsal corner, the rostral margin is ventrally oriented and forms a 90º angle with the dorsal orbital margin (1). ([20], character 123).
3. Inclusion of the prefrontal in the circumnarial fossa: absent (0); present (1). ([20], character 125).
4. Outward flaring of the rostrodorsal orbital margin of the prefrontal: absent, the prefrontal lies flush with the surrounding lacrimal and postorbital (0); present, the prefrontal flares dorsolaterally forming a thin and everted wing-like rim around the rostrodorsal margin of the orbit (1). ([20], character 126).

Postorbital

1. Dorsal surface of the postorbital above the jugal process: horizontal or slightly concave (0); deeply depressed (1). ([20], character 128, modified).
2. Rostrocaudal constriction of the dorsal region of the infratemporal fenestra: absent, caudal (squamosal) process of the postorbital elongate over the infratemporal fenestra (broad and subrectangular dorsal region of the fenestra) (0); present and caused by the presence of a nearly straight and oblique caudoventral margin of the caudodorsal region of the postorbital (dorsal region of infratemporal fenestra typically subtriangular) (1); present and caused by rostrocaudal shortening of the caudal process of the postorbital (dorsal region of infratemporal fenestra typically oval) (2). ([20], character 129).
3. Morphology of the central body of the postorbital: triangular, craniocaudally broad, expanded rostroventrally to form a straight and obliquely oriented caudodorsal orbital margin (0); triangular, with a caudodorsal orbital margin that ranges in lateral profile from semicircular to subsquared (1); rostrocaudally expanded, rostrally excavated and bulging laterally ('inflated'), containing a hollow inner cavity (in adults) (2). ([20], character 130).
4. Length of the jugal process of the postorbital: relatively short, approximately as long as the craniocaudal width of the orbit, hook-like in lateral profile (0); relatively long, longer than the craniocaudal width of the orbit, nearly straight, only slightly recurved rostrally (1). ([20], character 131).
5. Caudal extension of the caudal ramus of the postorbital that overlaps the laterodorsal surface of the squamosal: the caudal end of the postorbital caudal ramus extends caudodorsal to the precotyloid process, and over as much as the rostral half of the quadrate cotylus (0); the caudal end of the postorbital caudal ramus extends to a point rostral to the quadrate cotylus and does not overlap the latter (1). ([20], character 133, modified).

Squamosal

1. Length of the precotyloid process of the squamosal (measured as the ratio of its length relative to the width of the quadrate cotylus): precotyloid process distinctly longer than width of the quadrate cotylus (0); precotyloid process shorter than width of quadrate cotylus (1). ([20], character 134, modified).
2. Dorsoventral expansion of the caudolateral surface of the squamosal: unexpanded, shallowly exposed in caudal view (0); greatly expanded dorsomedially, forming a deep, near vertical, well-exposed face in caudal view (in adults) (1). ([20], character 135).
3. Separation of the squamosals at the occipital margin of the skull roof: completely separated by the parietal (0); the squamosal approach the sagittal plane of the skull, separated by a narrow band of parietal (1); extensive intersquamosal joint present at the midline, parietal completely excluded from the sagittal plane of the skull at that particular spot (in adults) (2). ([20], character 136).
4. Rostromedial indenture of the medial ramus of the squamosal: present, medial ramus of the squamosal curves rostromedially, so that the back of the skull appears to be deeply indented rostrally when viewed dorsally (0); absent, medial ramus of the squamosal extends medially, forming a subsquared caudolateral border of the skull roof (1). ([20], character 137).

Frontal

1. Bifurcation of the rostromedial margin of the frontals at the sagittal plane of the skull roof, leaving a V-shaped space in between: present (0); absent (1). ([20], character 138, modified).
2. Nasal articulation surface of the frontal shaped into a rostroventrally-slopping platform: absent (0); present (1). ([20], character 140).
3. Exposure of the frontal along the dorsal margin of the orbit: frontal exposed (0); frontal not exposed (1). ([20], character 143, modified).
4. Frontal upward doming dorsal to the braincase of subadult (and perhaps young adult) specimens: absent (0); present (1). ([20], character 144).

Parietal

1. Maximum length/minimum width proportions of the adult parietal: short, ratio between 1.40 and 2.35 (0); very short, length/width ratio less than 1.40 (1); relatively long, ratio greater than 2.35 (2). ([20], character 147, modified).
2. Orientation of the parietal midline crest: straight and level with the skull roof or slightly down-warped along its length (0); the sagittal crest deepens caudally and is strongly down-warped (1). ([20], character 148).
3. Morphology of the rostromedian process of the parietal that forms a crenulated suture in between the caudomedian margin of the frontals: rectangular, rostrocaudally short and mediolaterally expanded (0); rostrocaudally short and subtriangular to arcuate or absent (1); rostrocaudally elongate and mediolaterally narrow (2). ([20], character 149).
4. Rostral extension of the sagittal crest along the dorsal surface of the parietal: sagittal crest fades away or absent on the rostral third of the parietal (0); sagittal crest extends along the entire length of the parietal and remains sharp and well defined at the rostral region (1). ([20], character 150, modified).

Basioccipital

1. Length of basioccipital constriction: relatively long and well-developed (0); relatively short and poorly developed (1). ([20], character 153).

Basisphenoid

1. Orientation of the basipterygoid processes of the basisphenoid (measured as the angle between the ventral margins of both processes): angle less than 100° (0); angle of 100° or greater (1). ([20], character 154).
2. Developement of the alar process of the basisphenoid: moderately developed (0); very well developed, relatively large in size (1). ([20], character 155).
3. Development of the rostral constriction of the basisphenoid, caudal to the basipterygoid processes (measured as the ratio between the minimum mediolateral width of the rostral constriction and the maximum width of the basisphenoid across the sphenooccipital tubercles): relatively thick constriction, ratio less than 1.90 (0); very thin constriction, ratio greater than 1.90 (1). ([20], character 158, modified).

Laterosphenoid

1. Extreme reduction of the length of the postorbital process of the laterosphenoid to 25% or less the length of the mediodorsal flange of this element: absent (0); present (1). ([20], character 160).

Supraoccipital

1. Lateroventral corner of the supraoccipital deeply inset into the exoccipital, so that the latter is ‘locked’ between two short flanges that project medially above lateral end of the supraoccipital–exoccipital contact: absent (0); present (1). ([20], character 162).
2. Caudal extension of the exoccipital-supraoccipital shelf above the foramen magnum: very short rostrocaudal length, approximately less than half the diameter of the foramen magnum (0); moderately long, approximately more than half but less than the diameter of the foramen magnum (1); very long, substantially longer (often twice or more) than the diameter of the foramen magnum (2). ([20], character 163). Character treated as ordered.

Exoccipital-opisthotic

1. Orientation of caudal surface of paroccipital processes: faces mediocaudally (0); faces caudally (1). ([20], character 164, modified).

Palate

107. Ectopterygoid-jugal contact: present, the ectopterygoid contacts the medial side of the jugal (0); absent, the jugal lacks an articular facet for the ectopterygoid (1). ([20], character 167).

Regional cranial characters

108. Exposure of the nasal passage: present, nasal passage open and exposed on the lateral side of the rostrum (0); absent, nasal passage nearly or completely enclosed by bone and formation of internal cavities and passages (1). ([20], character 169).

109. Ratio between the length of the narial foramen and the distance between the rostroventral corner of the premaxilla and the rostroventral margin of the prefrontal: very short narial foramen, ratio up to 0.40 (0); moderately long narial foramen, ratio greater than 0.40 but less than 0.60 (1); elongated narial foramen, ratio between 0.60 and 0.65 (2). ([20], character 172).

110. Caudal extent of the nasal passage dorsal and/or caudal to the orbit: absent, nasal passage restricted to the antorbital region of the skull (0); present (1). ([20], character 176, modified).

111. Composition of the caudal margin of the functional external naris: formed by the nasal dorsally and the premaxilla ventrally (0); formed entirely by the nasal (1); formed entirely by the premaxilla (2). ([20], character 177).

112. Caudodorsal extension of the circumnarial fossa (homologous to the lateral diverticulum inside hollow supracranial crests): the fossa does not reach the caudal margin of the narial foramen and, thus, lacks a caudal margin (0); the fossa extends as far as to surround the caudal margin of the narial foramen, but does not reach the orbit (1); the fossa extends as far as the rostrodorsal region of the orbit (2); the fossa extends beyond the orbit, caudodorsal to its caudal margin (3). ([20], character 179).

113. Degree of excavation of the caudal region of the circumnarial fossa: lightly incised (0); deeply incised, but not invaginated in adults (1); deeply incised and invaginated in adults. ([20], character 180, modified). Character treated as ordered.

114. General shape of supracranial crest: absent (0); mediolaterally compressed arcuate protuberance, rostral or, in adults, dorsal to the level to the orbits (1); paddle-like and caudally (as well as slightly dorsally) directed solid blade of bone (2); mediolaterally narrow and paddle-like, extending caudal to the occiput (3); rostrally excavated and rostrally-facing protuberance (4); nasal fold that rises dorsally or caudodorsally to form a laterally excavated promontory (5); hollow supracranial crest (6). ([20], character 184, modified).

115. Palpebral (supraorbital) bone: present (0); absent (1). ([20], character 187, modified).

116. Length/width proportions of the orbit: nearly circular, approximately as wide as it is deep (0); elongated, dorsoventrally deeper than it is wide (1). ([20], character 188).

117. Shape and rostrocaudal width of the dorsal margin of the infratemporal fenestra relative to that of the dorsal margin: subrectangular, with a dorsal infratemporal margin that is approximately as wide as the ventral margin (0); subtriangular, with a dorsal infratemporal margin that is narrower than the ventral margin (1). ([20], character 191).

118. Location of the dorsal margin of the infratemporal fenestra relative to the dorsal margin of the orbit: the dorsal margin of the infratemporal fenestra lies approximately at the same level than the dorsal margin of the orbit and the caudal region of the skull roof is subhorizontal or slightly slopping rostroventrally relative to the frontal plane (0); the dorsal margin of the infratemporal fenestra is substantially more dorsally located than the dorsal margin of the orbit and the caudal region of the skull roof is rostroventrally inclined relative to the frontal plane (1); the dorsal margin of the infratemporal fenestra lies slightly or substantially below the level of the dorsal margin of the orbit and the caudal region of the skull roof is subhorizontal or slightly slopping caudoventrally relative to the frontal plane (2). ([20], character 192).

119. Morphology of the dorsal outline of the supratemporal fenestra: subrectangular, with the long axis directed rostrally (0); oval, with the long axis directed rostrolaterally (1). ([20], character 193, modified).

120. Maximum transverse width of the cranium in dorsal view across the postorbitals relative to the width across the quadrate cotylus of the squamosals: the skull is up to 25% wider across the postorbitals (0); the skull is more than 25% wider across the postorbitals (1). ([20], character 193, modified).

Vertebrae

121. Morphology of the dorsal flange of the axis: dorsally convex flange extending beyond or to the level of the cranialmost region of the postzygapophyses (0); presence of short cranial flange separated from the postzygapophyseal region by a prominent embayment (1). ([20], character 197).

122. Development of the postzygapophyseal processes of cranial and middle cervical vertebrae: relatively low and relatively short, less than three times the rostrocaudal breadth of the neural arch (0); relatively high and relatively long, three times or more longer than the breadth of the neural arch (1). ([20], character 198).

123. Height of the neural spine relative to that of the centrum of the tallest posterior dorsal or sacral vertebrae (in adults): relatively high neural spine, ratio greater than 2.10 (0); relatively low neural spine, ratio up to 2.10 (1). ([20], character 200, modified).

124. Slightly elongated neural spines in the cranial dorsal vertebrae, forming a 'wither-like' region above the pectoral girdle: absent (0); present (1). ([20], character 201).

125. Minimum count of co-ossified vertebrae in the sacral region (including single dorsal and caudal contributions: seven or fewer (0); eight or more (1). ([20], character 202).

Sternal

126. Length of the 'handle-like' caudolateral process of the sterna relative to that of the craniomedial plate (excluding the caudoventral process): caudolateral process slightly shorter or as long as the craniomedial plate (0); caudolateral process longer than the craniomedial plate (1). ([20], character 204).

Coracoid

127. Ratio between the length of the lateral margin of the facet for the scapular articulation and the length of the lateral margin of the glenoid: slightly longer scapular facet, ratio greater than 1 and up to 1.30 (0); glenoid longer than the scapular facet, with a ratio up to 1 (1). ([20], character 206, modified).

128. Angle between the lateral margins of the facet for scapular articulation and the glenoid: angle greater than 115º (0); angle up to 115º (1). ([20], character 207).

129. Morphology of the craniomedial margin of the coracoid: convex or straight, associated to a moderate development and slightly projected biceps tubercle (0); concave, associated to a relatively large and lateroventrally projected biceps tubercle (1). ([20], character 208).

130. Development of the 'hook-like' ventral process of the coracoid, measured as the ratio between the dorsoventral depth and the breadth of the process: relatively short, ratio less than 0.65 (0); relatively long, ratio more than 0.65 (1). ([20], character 209, modified).

131. Curvature of the ventral hook-like process of the coracoid: ventrally directed (0); recurved, so that the process is caudoventrally directed (1). ([20], character 210).

Scapula

132. Lateral profile of the dorsal margin of the scapula: craniocaudally straight from the cranial margin of the coracoid facet to the distal end of the blade (0); curved, dorsally convex, curvature originating at the level of the dorsal margin of the pseudacromial process, and most pronounced over the dorsoventral constriction (1). ([20], character 211).

133. Scapular length, ratio between the craniocaudal length of the scapula (from the cranial end of the acromion process to the distal margin of the blade) and the dorsoventral depth of the cranial end (from the cranial end of the acromion process to the ventral apex of the glenoidal facet): relatively short scapula, ratio up to 4 (0); relatively long scapula, ratio greater than 4 (1). ([20], character 212).

134. Dorsoventral expansion of the distal region of the scapular blade (measured as a ratio between the depth of the distal end of the blade and the depth of the proximal region): ratio less than 1 (0); ratio of 1 or greater (1). ([20], character 213).

135. Proximal constriction (scapular 'neck'), ratio between the dorsoventral width of the proximal constriction and the dorsoventral depth of the cranial end of the scapula: narrow 'neck', ratio up to 0.60 (0); relatively broad 'neck', ratio greater than 0.60 (1). ([20], character 214).

136. Morphology and orientation of the pseudoacromial process of the scapula: recurved, so that the cranial region is dorsally or craniodorsally directed (0); horizontal, occasionally with minor and subtle dorsal or ventral curvatures, so that the cranial region is cranially or mostly cranially directed (1). ([20], character 215).

137. Cranial extension of the craniodorsal region of the scapula (bearing the coracoid facet), measured as a ratio between the distance from the coracoid joint and the cranial end of the pseudoacromial process and the height between this and the ventral apex of the glenoidal facet: short craniodorsal region, ratio less than 0.45 (0); long craniodorsal region, ratio of 0.45 or greater (1). ([20], character 217).

138. Development of the deltoid ridge: dorsoventrally narrow convexity limited to the proximal region of the scapula, near the pseudoacromial process from which it develops, with a poorly demarcated ventral margin (0); dorsoventrally deep and craniocaudally long, with a well demarcated ventral margin (1). ([20], character 218).

Humerus

139. Length of the deltopectoral crest of the humerus (measured as the ratio between the proximodistal length of the crest and the proximodistal length of the humerus): proximodistally short crest, ratio less than 0.48 (0); ratio between 0.48 and 0.55 (1); very long crest, ratio greater than 0.55 (2). ([20], character 219).

140. Lateroventral expansion of the deltopectoral crest of the humerus (measured as the ratio between the width of the humerus across the distal fourth of the deltopectoral crest and the width of the distal shaft at the point of maximum curvature): poorly expanded deltopectoral crest, ratio less than 1.65 (0); ratio between 1.65 and 1.90 (1); very expanded deltopectoral crest, ratio greater than 1.90 (2). ([20], character 220).

141. Degree of angulation of the ventral margin of the deltopectoral crest: well-rounded (0); extending abruptly from the humeral shaft to give a distinct angular profile (1). ([20], character 221).

142. Overall proportions of the humerus (measured as the ratio between the total length and the width of the lateral surface of the proximal end of the humerus: ratio between 4.25 and 4.90 (0); relatively short and stocky humerus, ratio less than 4.25 (1); relatively long and thin humerus, ratio greater than 4.90 (2). ([20], character 222, modified).

Ulna

143. Length of the ulna relative to its dorsoventral thickness (measured at mid-shaft): ratio length/width less than 10 (0); ratio length/width equal or larger than 10 (1). ([20], character 223).

Manus

144. Manual digit I: presence of metacarpal I and one ungual phalanx (0); entire digit I absent (1). ([20], character 226).

Ilium

145. Angle of ventral deflection of the preacetabular process: angle greater than 150º (0); angle of 150º or less (1). ([20], character 232).

146. Dorsoventral depth of the proximal region of the preacetabular process (measured as a ratio between this and the dorsoventral distance between the pubic peduncle and the dorsal margin of the ilium): shallow, less than half the depth of the cranial central blade, ratio less than 0.50 (0); approximately as deep as the cranial central blade depth, ratio between 0.50 and 0.55 (1); deeper than half the depth of the cranial central blade, ratio greater than 0.55 (2). ([20], character 233).

147. Dorsoventral depth of the central blade (expressed as a ratio between this and the distance between the pubic peduncle and the caudodorsal prominence of the ischial peduncle): ratio of 0.80 or greater (0); ratio less than 0.80 (1). ([20], character 234).

148. Position of the ventralmost margin of the supraacetabular process relative to the caudoventral margin of the lateral ridge of caudal protuberance of the ischial peduncle: apex located caudodorsally (0); apex located craniodorsally (1). ([20], character 235).

149. Development of the lateroventral projection of the supraacetabular process: forms a longitudinal and continuous 'swelling' or reflected border along the dorsal margin of the central blade and the proximal region of the postacetabular process, with a depth up to 25% the depth of the ilium (0); projected lateroventrally at least 25% (but less than half) the depth of the ilium (1); projects lateroventrally between half and three quarters of the dorsoventral depth of the ilium (2); projects lateroventrally to overlap totally or at least half of the lateral ridge of the caudal prominence of the ischial peduncle (3). ([20], character 236).

150. Symmetry of the lateral profile of the supraacetabular process: asymmetrical, with a caudally skewed lateral profile (0); symmetrical or with a slightly caudally skewed profile (1). ([20], character 238).

151. Morphology of the lateroventral margin of the supraacetabular process: craniocaudally sinuous (0); widely arched (1); U-or V- shaped (2); subrectangular, with a shallow notch that divides the ventral margin in two poorly demarcated lobes (3). ([20], character 239).

152. Morphology of the ischial peduncle: relatively large and dorsoventrally deep (longer than wide), subconical, with a proximal region that is only slightly craniocaudally wider than the distal end of the process (0); relatively shorter (wider than or as wide as long) and triangular, with a proximal region that is much craniocaudally wider than the distal end (1). ([20], character 241).

153. Morphology of the ischial peduncle: formed by a single and large, oval ventral protrusion (0); composed of a large and oval ventral protrusion and by a smaller, caudodorsally located prominence emerging from the caudodorsal ridge (1); formed by two protrusions of similar size, the caudalmost one located slighty caudodorsally (2). ([20], character 242).

154. Ratio between the craniocaudal length of the postacetabular process and the craniocaudal length of the central blade of the ilium: short postacetabular process, ratio up to 0.80 (0); postacetabular process nearly as long as the central plate, ratio greater than 0.80 but less than 1.1 (1); postacetabular process substantially longer than the central plate, ratio of 1.1 or greater (2). ([20], character 243).

155. Brevis shelf at the base of the postacetabular process: present (0); absent (1). ([20], character 244, modified).

156. Geometry of the lateral profile of the postacetabular process: the ventral margin converges caudodorsally to meet the horizontal dorsal margin, forming a tapering caudal end and producing a triangular lateral profile of the process (0); dorsal and ventral margins parallel or slightly convergent, forming a distinct (rectangular or subcircular) caudal margin (1). ([20], character 247).

157. Orientation of the dorsal margin of the postacetabular process relative to the acetabular margin: horizontal dorsal margin, parallel or nearly parallel to the acetabular margin (0); caudodorsally oriented dorsal margin, rising dorsally relative to acetabular margin (1). ([20], character 248).

Pubis

158. Orientation of the dorsoventral expansion of the prepubic process: the dorsal region of the expansion is more expanded than the ventral region, so that distally the process is dorsally directed (0); the ventral region is more expanded than the dorsal region, so that the distal expansion is ventrally directed (1). ([20], character 252).

159. Geometry of the dorsoventral expansion of the prepubic process (in lateral or medial views): circular to oval expansion, extensive and convex ventral margin (0); subsquared distal dorsal margin, expansion dorsoventrally taller than cranioventrally long, very pronounced proximal dorsal concavity and nearly straight distal ventral margin (1); ellipsoidal, expansion craniocaudally longer than dorsoventrally tall, well-pronounced concavities of the dorsal and ventral proximal margins (2); oval expansion, dorsoventrally taller than craniocaudally long, well-pronounced concave profiles of dorsal and ventral proximal margins (3); rectangular, craniocaudally longer than dorsoventrally tall, nearly straight profiles of the dorsal and ventral proximal margins (4). ([20], character 253).

160. Craniocaudal length of the proximal constriction of the prepubic process of the pubis relative to length of the dorsoventral expansion: constriction slightly shorter than the dorsoventral expansion, which begins at the proximal region of the process (0); constriction and distal expansion have approximately the same length (1); constriction longer than the dorsoventral expansion, which is restricted to the distal region of the process (2). ([20], character 255, modified).

161. Relative position of maximum concavity of the dorsal and ventral margins of the prepubic process: maximum ventral concavity achieved adjacent to the proximal region of the postpubic process, maximum dorsal concavity located further distally (0); maximum ventral concavity located ventral to or slightly caudal to the maximum dorsal concavity (1). ([20], character 256).

162. Total length of the pubis, as the ratio between the craniocaudal distance from the acetabular margin to the distal margin of the prepubic process and the distance from the dorsal margin of the iliac peduncle and the ventral margin of the proximal postpubic shaft: short, ratio less than 3 (0); long, ratio greater than 3 (1). ([20], character 262, modified).

Ischium

163. Development of a caudal curvature of the distal margin of the iliac peduncle: absent or faintly developed (0); presence of a well-developed curvature in the caudodorsal corner, so that the peduncle appears 'thumb-like' in lateral and medial profiles (1). ([20], character 263, modified).

164. Elongation of the iliac peduncle of the ishium (ratio between the proximodistal length and the craniocaudal width of the distal margin): relatively short peduncle, ratio less than 2 (0); relatively long peduncle, ratio greater than 2 (1). ([20], character 264, modified).

165. Relative orientation of the acetabular and caudodorsal margins of the iliac peduncle of the ischium: margins are either parallel or slightly convergent relative to each other (correlated with a greater expansion of the craniodorsal corner of the peduncle) (0); margins become slightly to greatly divergent near the proximal region of the peduncle (1). ([20], character 266).

166. Orientation of the craniocaudal axis of the pubic peduncle (perpendicular to its articular margin) relative to the ischial shaft: ventrally inclined, angle up to 130º (0); slightly inclined ventrally or parallel, angle greater than 130° (1). ([20], character 267, modified).

167. Length/width proportions of the pubic peduncle: approximately as long or slightly longer proximodistally as the distal articular surface is dorsoventrally wide (0); proximodistally shorter than the dorsoventral width of the distal articular surface (1). ([20], character 268, modified).

168. Relative position of the dorsal acetabular margin of the pubic peduncle: ventral to or at the same level as the dorsal margin of the ischial shaft (0); peduncular margin set dorsal to the dorsal margin of the ischial shaft (1). ([20], character 269).

169. Dorsoventral thickness of the mid-shaft of the ischium (measured as a ratio between this and the length of the entire shaft): relatively thick shaft, more than 5% the length of the ischial shaft (0); very thin shaft, up to 5% the length of the ischial shaft (1). ([20], character 270, modified).

170. Morphology of the distal region of the ischial shaft: ventrally expanded, forming a large 'foot' or 'boot-like' process (0); slightly expanded into a blunt end (1). ([20], character 271, modified).

Femur

171. Degree of curvature of the distal half of the femoral shaft: slightly curved caudomedially (0); absence of curvature, straight distal shaft (1). ([20], character 275).

172. Lateral profile of the caudoventral margin of the fourth trochanter: triangular and ending in a caudally, and slightly ventrally, directed point (0); smooth and arcuate (1). ([20], character 276).

Pes

173. Length/width proportions of metatarsal III (measured as the ratio between its proximodistal length and its mediolateral breadth at mid-shaft): elongated, ratio of 4.50 or greater (0); relatively short, ratio less than 4.50 (1). ([20], character 282, modified).

174. Length/width proportions of the disc-shaped pedal phalanges III2-III3: up to three times (or less) wider than they are proximodistally long (0); more than three times wider than they are proximodistally long (1). ([20], character 284).

175. Morphology of the pedal unguals: proximodistally elongated and arrow-shaped, with a bluntly truncated tip and prominent claw grooves (0); mediolaterally broad and proximodistally shortened, rounded shield or hoof-like shaped, with reduced or absent claw grooves (1). ([20], character 285).

176. Ridge on the plantar surface of pedal unguals: absent (0); present (1). ([20], character 286).
