# Supplementary material for: A New Saurolophine Dinosaur from the Latest Cretaceous of Far Eastern Russia
Source: PLoS One. 2012 May 30;7(5):e36849. doi: 10.1371/journal.pone.0036849 (PMC3364265; doi:10.1371/journal.pone.0036849)
Supplement: Text S2 — Tree description of the larger cladogram ( Fig. 20 ). (DOCX) [file pone.0036849.s004.docx]

**Text S2: Tree description of the larger cladogram (Fig. 20)**

Character transformations were evaluated under unambiguous, fast and slow optimisation options in Winclada [40]; unambiguous synapomorphies are those that diagnose a node under both fast and slow optimisations. Node numbers refer to Fig. 20. The synapomorphies supporting each clade are indicated by a pair of numbers. The number to the left of the dash is the character number corresponding in the list in Appendix 2, whereas the number between brackets represents the character state. Numbers in bold are unambiguous and unequivocal (CI = 1) synapomorphies.

**Node A (Hadrosauridae):** Unambiguous: 1 (1), **4 (1)**, **5 (1)**, **65 (2)**, **77 (1)**, **90 (1)**, **106 (1)**, **114 (5)**, **143 (1)**, **148 (1)**, **149 (2)**, **153 (2)**, **156 (1)**; Fast: 3 (1), 10 (1), 11 (1), 13 (1), 17 (1), 18 (1), 19 (1), 20 (1), 22 (1), 25 (1), 26 (1), 28 (1), 29 (1), 30 (1), 31 (1), 32 (1), 34 (1), 36 (1), 40 (1), 41 (1), 51 (1), 56 (1), 58 (2), 59 (1), 60 (1), 61 (1), 63 (1), 64 (1), 68 (2), 73 (1), 75 (1), 97 (1), 99 (1), 100( 1), 104 (1), 111 (1), 112 (1), 115(1), 116 (1), 117 (1), 122 (1), 125 (1), 128 (1), 129 (1), 130 (1), 131 (1), 132 (1), 133 (1), 135 (1), 150(1), 151 (2), 154 (1), 155 (1), 158 (1), 160(1), 161 (1), 170 (1).

**Node B:** Unambiguous: 8 (1); Fast: 175 (0); Slow: 170 (1).

**Node C (Saurolophidae):** Unambiguous: **2 (1)**, **9 (1)**, **67 (1)**, **70 (1)**, **84 (1)**, **141 (1)**, **157 (1)**, **167 (1)**; Fast: 6 (1), 89 (1), 112 (2), 139 (1), 140 (2); Slow: 3 (1), 10 (1), 11 (1), 13 (1), 14 (1), 18 (1), 20 (1), 22 (1), 25 (1), 26 (1), 28 (1), 29 (1), 30 (1), 31 (1), 32 (1), 34 (1), 36 (1), 40 (1), 58 (2), 59 (1), 60 (1), 63 (1), 64 (1), 73 (1), 97 (1), 99 (1), 100 (1), 104 (1), 115 (1), 116 (1), 122 (1), 15 (1), 129 (1), 130 (1), 131 (1), 132 (1), 133 (1), 135 (1), 144 (1), 151 (2), 155 (1), 158 (1), 161 (1).

**Node D (Lambeosaurinae):** Unambiguous: 16 (1), **47 (1)**, **49 (1)**, **55 (1)**, **57 (1)**, **66 (2)**, 74 (1), **75 (2)**, **78 (1)**, 81 (1), 82 (1), **88 (1)**, 92 (1), 93 (1), **94 (1)**, **95 (1)**, 96 (1), **108 (1)**, **110 (1)**, **111 (2)**, **114 (6)**, **118 (2)**, **119 (1)**, 124 (1), 139 (2), 142 (1), **163 (1)**; Fast: 12 (1), 17 (2), 56 (0), 61 (2), 68 (3), 71 (0), 76 (1), 138 (0), 149 (1), 160 (2), 170 (0); Slow: 41 (1), 68 (3), 140 (2), 150 (1).

**Node E (Saurolophinae):** Unambiguous: **105 (1)**, **136 (1)**; Fast: 4 (2), 37 (1), 39 (2), 41 (3), 43 (1), 45 (1), 72 (1), 79 (1), 85 (1), 126 (1), 159 (2), 168 (1), 169 (1); Slow: 61 (1), 75 (1), 138 (1), 139 (1), 170 (1).

**Node F:** Unambiguous: **20 (2)**, **98 (1)**; Fast: 140 (0); Slow: 17 (1), 37 (1), 39 (2), 41 (3), 43 (1), 45 (1), 51 (1), 56 (1), 68 (2), 85 (1), 111 (1), 112 (2), 126 (1), 128 (1), 160 (1), 168 (1), 175 (1).

**Node G (Brachylophosaurini):** Unambiguous: 91 (1), **101 (1)**, **102 (1)**, 120 (1); Fast: 12(2), 16 (2), 21 (1), 50 (1), 62 (1), 89 (2), 114 (2), 121 (1), 150 (0), 151 (3), 169 (0), 176 (1); Slow: 4 (2), 6 (1), 71 (1), 72 (1), 89 (2), 117 (1).

**Node H:** Unambiguous: **27 (1)**, 81 (1), **87 (1)**, 95 (2); Fast: 79 (0); Slow: 12 (2), 16 (2), 19 (1), 50 (1), 62 (1), 121 (1), 151 (3), 154 (1), 159 (1), 176 (1).

**Node I:** Unambiguous: **53 (1)**, **66 (1)**, **69 (1)**, **72 (2)**, **105 (2)**; Fast: 6 (0), 7 (1), 9 (2), 86 (1), 117 (0), 118 (1), 123 (1), 137 (1), 140 (1), 147 (1) 154 (2), 159 (4), 162 (1), 173 (1), 174 (1); Slow: 79 (1).

**Node J:** Unambiguous: **2 (2)**, 14 (0), 17 (3), 95 (2); Fast: 71 (0), 112 (1); Slow: 4 (2); 89 (1); 126 (1), 137 (1), 140 (1), 147 (1), 150 (1), 154 (2), 159 (4), 162 (1).

**Node K (*Gryposaurus*):** Unambiguous: **41 (2)**, **52 (0)**, **56 (2)**, **114 (1)**, 133 (0), 134 (0), **142 (2)**, **146 (1)**; Fast: 9 (1), 19 (0), 48 (1), 53 (2), 54 (1), 75 (0), 97 (0), 109 (3); Slow: 7 (1), 112 (1).

**Node L:** Unambiguous: 39 (1); Fast: 54 (2); Slow: 48 (1), 53 (2), 54 (2), 75 (0), 86 (1), 97 (0), 109 (3), 118 (1).

**Node M:** Unambiguous: **1 (2)**, **23 (1)**, **24 (1)**, **50 (2)**, **51 (2)**, **68 (1)**, 76 (1), 83 (1), **113 (1)**, 117 (1), **164 (1)**; Fast: 86 (0), 118 (0); Slow: 9 (2), 19 (1), 74 (1).

**Node N (Saurolophini):** Unambiguous: 41 (1), 73 (0), 80 (1), 81 (1), 93 (1), **109 (2)**, 113 (2); Fast: 112 (2), 121 (1), 179 (3), 169 (0); Slow: 7 (1), 174 (1).

**Node O (*Saurolophus*):** Unambiguous: 39 (1), 53 (0), 79 (0), 82 (1), 83 (2), 89 (2), 112 (3), **114 (3)**, 124 (1), 140 (2), 142 (1), 143 (0), 162 (0); Fast: 139 (2), 168 (0); Slow: 121 (1); 149 (3); 173 (1)

**Node P (Edmontosaurini):** Unambiguous: 114 (0), 154 (1), 160 (2); Fast: 7 (0), 12 (1), 16 (1), 20 (3), 44 (1), 46 (1), 62 (1), 85 (0), 97 (2), 120 (1), 159 (2), 174 (0); Slow: 112 (1), 169 (1).

**Node Q:** Fast: 53 (2), 82 (1), 134 (0), 142 (1), 145 (0).

**Node R (*Edmontosaurus*):** Unambiguous: 73 (2), **84 (2)**, **103 (1)**, 113 (2); 137 (0), 139 (2), **159 (3)**; Slow: 12 (1), 16 (1), 20 (3), 44 (1), 46 (1), 62 (1), 85 (0), 97 (2), 120 (1).
